# Bottom‐Up Synthesis and Purification of Extracellular Vesicle Mimetics

**DOI:** 10.1002/jev2.70190

**Published:** 2025-11-18

**Authors:** Luuk van de Schepop, You Lin Stiemsma, Lin Xia Wichers, Emma S. Hotting, Simone Smits, Cornelis W. Seinen, Tanja V. Edelbacher, Arjan D. Barendrecht, Annet C. W. van Wesel, Raymond M. Schiffelers, Pieter Vader

**Affiliations:** ^1^ CDL Research University Medical Center Utrecht Utrecht The Netherlands; ^2^ Department of Experimental Cardiology University Medical Center Utrecht Utrecht The Netherlands

**Keywords:** cell‐free protein synthesis, drug delivery, extracellular vesicles, liposomes, mimetics, synthetic nanoparticles

## Abstract

Extracellular vesicles (EVs) are nanosized particles secreted by all cell types. As EVs are naturally occurring carriers of biological cargo, they serve as a promising candidate for drug delivery applications. Potential advantages of EVs as drug delivery systems include biological stability, intrinsic targeting properties and ability to overcome natural barriers. However, limitations such as cumbersome production and isolation procedures, batch‐to‐batch variability, and challenges related to efficient cargo loading limit their potential for clinical applications. Here, we introduce EV mimetics, prepared by incorporating full‐length membrane proteins in the lipid bilayer of liposomes, using cell‐free protein synthesis. These structures mimic functional characteristics of EVs, while offering advantages in terms of ease of manufacture, controllability and potential for efficient cargo loading. To demonstrate the feasibility of producing proteoliposomes as EV mimetics, we selected EV‐associated CD47, CD39 and N‐Cadherin as model proteins. We show successful production and purification of CD47, CD39 and N‐Cadherin containing EV mimetics. Additionally, for CD47, we show that reaction conditions can be tailored to enhance EV mimetic yield. Furthermore, proteinase K protection assays and immuno‐labelling electron microscopy revealed that correct membrane protein topology is preserved for CD47 and CD39. N‐Cadherin EV mimetics show enhanced uptake by N‐Cadherin‐expressing MDA‐MB‐231 cells, proving membrane protein functionality is preserved. We demonstrate the versatility of the methodology by producing EV mimetics using a wide variety of liposomal formulations. Finally, we show that two distinct membrane proteins can be inserted in the same EV mimetic, further indicating versatility and broad applicability. This study presents a modular and controllable strategy for cell‐free synthesis of functional EV mimetics, which provides a meaningful step toward addressing challenges in EV‐inspired drug delivery development.

## 1 Introduction

Extracellular vesicles (EVs) are nanosized (30–1000 nm) lipid bilayer enclosed particles secreted by all cell types (Elsharkasy et al. 2020). EVs are a collective term for a wide range of vesicular nanostructures including exosomes, microvesicles (or ectosomes) and apoptotic bodies (Ginini et al. 2022). Amongst many functions, EVs are recognized as mediators of cell–cell communication (Ginini et al. 2022). EVs can vary in size and contain biological cargo such as nucleic acids, proteins and metabolites (Kumar et al. 2024). As EVs are naturally occurring carriers of such cargo, they are increasingly being considered as a promising candidate for drug delivery applications (Elsharkasy et al. 2020). Potential benefits of EVs as drug delivery systems include enhanced drug delivery capacity, partially due to their ability to cross biological barriers, intrinsic cell specific targeting capacity and benefits related to biocompatibility (Elsharkasy et al. 2020; Bader et al. 2024; Murphy et al. 2021).

However, translation of EVs to clinical drug delivery applications remains challenging. EVs are generally produced by in vitro cell cultures and subsequently purified. However, their physiochemical properties—such as their broad size distribution and their presence in complex biological fluids—form a challenge for appropriate purification (Du et al. 2023). As a result, purification procedures are often cumbersome and result in low yields. Additionally, changes in conditions during in vitro cell culture result in differences in biological content of EVs, in turn resulting in batch‐to‐batch variability (Korchak et al. 2023). Moreover, each purified batch of EVs in itself comprises a heterogeneous mixture of EVs with different biological origin (Wang et al. 2023). Another significant challenge is efficient loading of biological cargo into EVs (de Jong et al. 2019). Efforts have been made to load EVs by overexpressing cargo in the parental cell, or by techniques aiming to load cargo after formation of EVs such as electroporation or freeze‐thawing. However, these approaches often suffer from low or inconsistent loading efficiencies, and some can compromise EV integrity (Elsharkasy et al. 2020; Joshi et al. 2021).

To improve upon the aforementioned issues regarding EVs as drug delivery systems, we here aimed to develop EV mimetics. In this work, EV mimetics are defined as proteoliposomes containing full‐length membrane proteins. Liposomes, synthetic nanoparticles composed of an aqueous core enclosed by a phospholipid bilayer, share structural similarities with EVs. Liposomes are easy to produce with tunable physiochemical properties such as size, concentration and charge. Furthermore, liposomes allow for the efficient loading of biological cargo, such as RNA and proteins, with high yield (Pande 2023; Nsairat et al. 2023). Notably, liposomal drug delivery systems have already been approved for clinical use (Liu et al. 2022; Nsairat et al. 2022).

Here, we set out to develop a strategy to synthesize EV mimetics with controlled composition and functionality. We describe a cell‐free protein synthesis‐based approach for the production and purification of EV mimetics incorporating CD47, CD39 or N‐Cadherin. We tailored several reaction conditions to optimize yield. We characterized membrane proteins by assessing their protein topology, as well as by testing membrane protein functionality upon incorporation in liposomes. We further investigated the versatility of this methodology by employing a diverse range of liposomal formulations for EV mimetic production. Finally, we investigated the feasibility of incorporating multiple membrane proteins into a single EV mimetic.

## 2 Materials and Methods

### 2.1 Materials

PURExpress in vitro protein synthesis kit, disulfide bond enhancers, murine RNase inhibitor and DHFR plasmids were purchased from New England Biolabs. All oligonucleotides were ordered at Integrated DNA Technologies. 1,2‐Dioleoyl‐sn‐Glycero‐3‐Phosphatidylcholine (DOPC), 1,2‐distearoyl‐sn‐glycero‐3‐ phosphoethanolamine‐N‐(Cyanine 5) (18:0 Cy5 PE), 18:1 (Δ9‐Cis) PE (DOPE), 1,2‐distearoyl‐sn‐glycero‐3‐phosphoethanolamine‐N‐[methoxy(polyethylene glycol)‐2000] (DSPE‐PEG‐2000), were purchased from Avanti Polar Lipids. Cholesterol was purchased from Sigma. Strep‐Tactin XT resin and BXT buffer were purchased from IBA‐Lifesciences. 15 nm ProteinA gold, Uranyl acetate and uranyl acetate methyl cellulose were prepared and provided by the Department of Cell Biology at the UMCU.

### 2.2 Construct Cloning

gBlocks were ligated into pJET1.2/Blunt cloning vectors using a CloneJET PCR cloning kit (Thermo Fisher Scientific). Next, vectors were transformed into TOP10 *Escherichia coli* cells, allowed to grow and plasmid DNA was subsequently purified using the NucleoSpin Plasmid kit (NEB). gBlocks were digested using restriction enzymes NdeI and BamHI (NEB) and cloned into DHFR plasmids. To this end, a Quick Ligation Kit (NEB) was used. Again, plasmid was transformed into TOP10 *E. coli* cells and cells were allowed to grow. Then, plasmid was purified using a NucleoSpin Plasmid Kit (NEB). Plasmids were sequenced to rule out any mutations using Sanger sequencing (Macrogen). Plasmid concentration was analysed using a DS‐11 Fluorometer Spectrometer (DeNovix). All final plasmid sequences in DHFR vectors used are listed in Table S1.

### 2.3 Liposome Preparation

Liposomes were prepared using the thin film hydration method. Briefly, stock solutions of lipids were prepared in either ethanol (for DOPC, DOPE, Cholesterol) or chloroform (for DOPS, 18:0Cy5PE), and added to a 50 mL round‐bottom flask. The flask was continuously rotated under reduced pressure in a 55°C water bath using a rotary evaporator (Büchi Labortechnik) until a dry lipid film was obtained. The film was then dried for 10 min under flowing nitrogen gas to remove traces of solvent. Subsequently, the lipid mix was hydrated using HBS (10 mM HEPES and 150 mM NaCl in dH2O, pH 7.4) in the same rotary setup at 55°C. Glass beads were added to enhance mixing. The liposome solution was then extruded 11 times using an Avanti hand extruder (Avanti Polar Lipids), through a Whatman 100 nm polycarbonate membrane (Cytiva). Liposomes were stored in the dark at 4°C.

### 2.4 Lipid Quantification

Quantification of phospholipids was determined using a Rouser assay (Rouser et al. 1966). A sodium dihydrogen phosphate monohydrate (0.5 mM) standard was prepared in dH2O, and stored at 4°C. The standard was added to glass tubes in steps of 10 nmol up to 80 nmol. A sample of liposomes was added to a clean glass tube. The solvent was evaporated by incubation in a heating block at 180°C for 20 min. Subsequently, tubes were cooled to RT, and 300 µL perchloric acid (70%) was added to each tube. Tubes were closed to prevent evaporation, and incubated at 180°C for 45 min. Tubes were cooled to RT, and mixed with 1000 µL dH2O, 500 µL ammonium heptamolybdate solution (1.25% in dH2O; 10.7 mM) and 500 µL ascorbic acid (5% in dH2O; 284 mM). Upon mixing, tubes were closed again and incubated in a 70°C water bath for 5 min. Tubes were cooled to RT, and 150 µL was pipetted into a transparent 96‐well F‐bottom microplate in triplo. Absorbance was measured at 797 nm using the SpectraMax iD3 microplate reader and SoftMax Pro software (Molecular Devices).

### 2.5 Cell‐Free Protein Synthesis

Cell‐free protein synthesis was performed using the PURExpress in vitro protein synthesis kit. Solution A and B of the PURExpress in vitro protein synthesis kit were stored at −80°C and thawed on ice right before use. PURExpress disulfide bond enhancers, murine RNase inhibitor and plasmid DNA templates were stored at −20°C and thawed on ice right before use. Liposomes were stored at 4°C. Cell‐free protein synthesis were prepared by mixing 40 µL component A, 30 µL component B, 4 µL PURExpress disulfide bond enhancer I, 4 µL PURExpress disulfide bond enhancer II, 2 µL murine RNase inhibitor, 1 µg plasmid DNA template and liposomes to obtain a final concentration of 5 mM (except where noted otherwise). If needed, volume was supplemented with HBS to a final volume of 100 µL. Reaction mixes were carefully resuspended and incubated for 4 h or overnight at 37°C (except where noted otherwise) in an air incubator. Mixes were subsequently stored at 4°C in the dark. For experiments where liposomes were added post‐incubation, HBS was added instead of liposomes. Following incubation, liposomes were added to obtain a final lipid concentration of 5 mM and incubated for 10 min at 37°C before further use.

### 2.6 Density Gradient Ultracentrifugation

**FIGURE 1:**
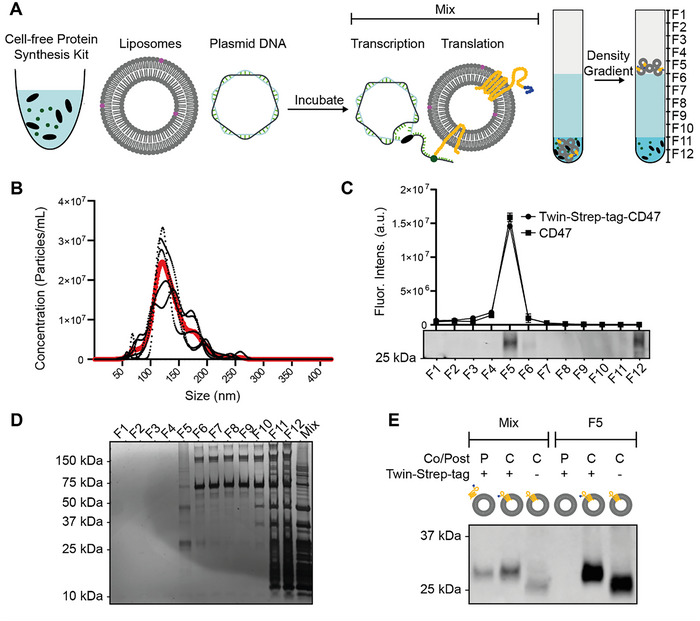
Co‐incubation of in vitro cell‐free protein synthesis components, plasmid DNA and liposomes allows successful production of CD47 EV mimetics. (A) Schematic overview of the reaction performed, followed by sucrose density gradient ultracentrifugation (DGU) to isolate EV mimetics. (B) Nanoparticle Tracking Analysis for DOPC/18:0Cy5PE (99.8/0.2) liposomes. Each black dotted line represents a single measurement, whereas the red line represents the mean of all measurements. (C) Fluorescent lipid quantification, indicating distribution of liposomes following DGU. Furthermore, a Strep‐tag labelled western blot of all fractions upon DGU of Twin‐Strep‐tag‐CD47 EV mimetics is shown. (D) Silver stain analysis of cell‐free protein synthesis reaction and all fractions upon DGU of Twin‐Strep‐tag‐CD47 EV mimetics. E: CD47 labelled western blot of cell‐free protein synthesis reactions performed when liposomes were added before (co) or after (post) coupled transcription/translation of protein. Samples before and after DGU (Mix and F5, respectively) are shown.

**FIGURE 2:**
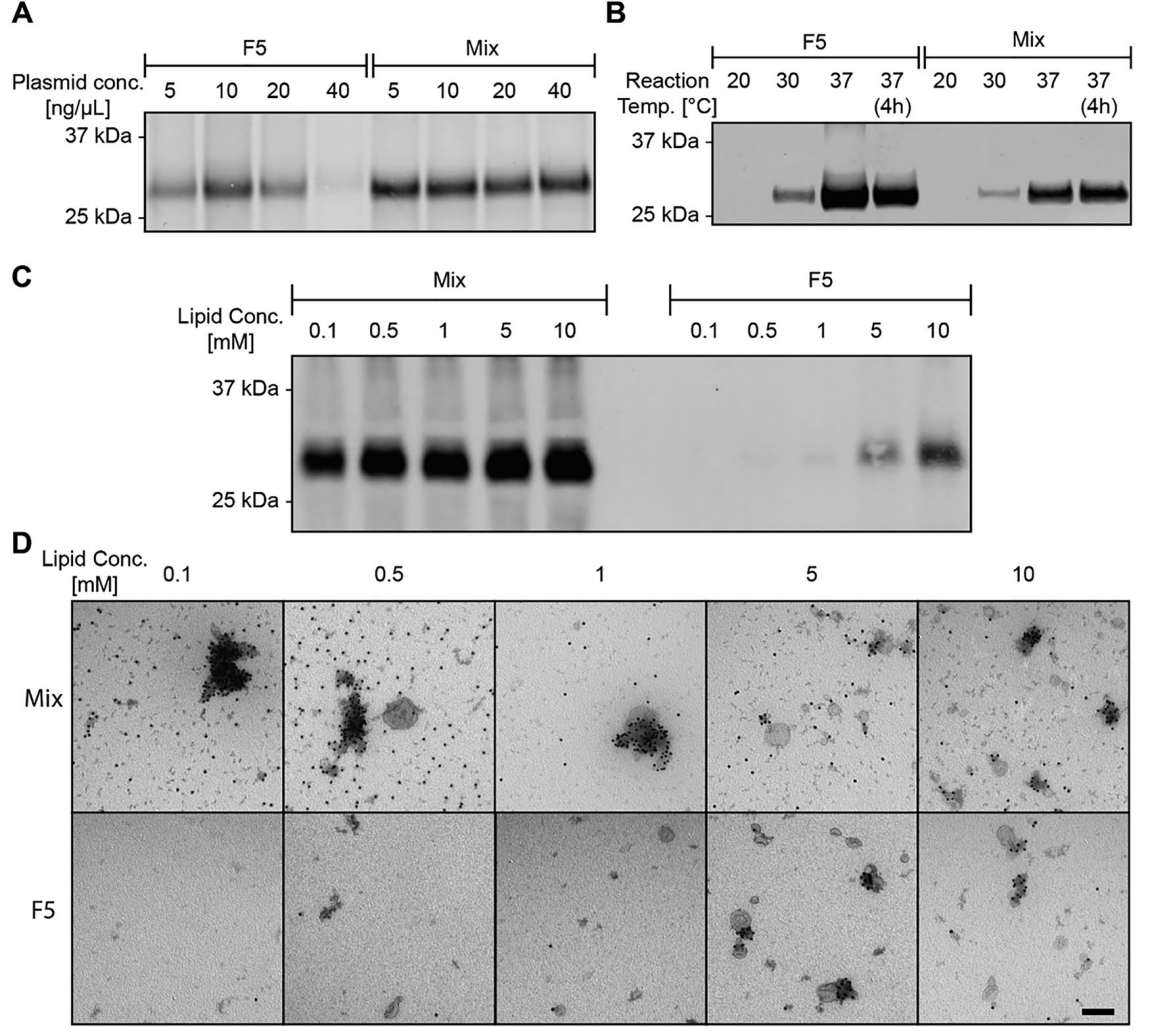
Optimization of reaction conditions allows for enhanced EV mimetic yield. (A) Strep‐tag labelled western blot of EV mimetics for plasmid concentration optimization for Twin‐Strep‐tag‐CD47, both directly upon synthesis (Mix) and upon density gradient ultracentrifugation (DGU), labelled Fraction 5 (F5). (B) Strep‐tag labelled western blot of EV mimetics for reaction temperature optimization, both directly upon synthesis (Mix) and upon DGU (F5). (C) Strep‐tag labelled western blot of EV mimetics for lipid concentration optimization, both directly upon synthesis (Mix) and upon DGU (F5). (D) Strep‐tag labelled immuno‐electron microscopy images of EV mimetics, both directly upon synthesis (Mix) and upon DGU (F5). Scale bar: 200 nm.

First, a stock solution of 40% sucrose in HBS was prepared, and sterile filtered (0.45 µm). 0.2% BSA and 1X cOmplete protease inhibitor cocktail (PIC, Roche) was supplemented to the 40% sucrose solution. Furthermore, a 0% sucrose solution was prepared by supplementing 1X HBS with PIC and 0.2% BSA. All solutions were sterile filtered (0.45 µm). For the following figures, sucrose solutions were prepared without PIC and BSA supplementation: Figures 1, 2, 4D, E and S2B. 12 mL density gradient ultracentrifugation (DGU) tubes were prepared by mixing 90 µL cell‐free protein synthesis mix with 1.91 mL 40% sucrose. Subsequently, 5 mL 25% sucrose was carefully layered on top, followed by 5 mL 0% sucrose. Tubes were balanced with 0% sucrose solution if necessary. Then, tubes were centrifuged in a SW40Ti swing‐out rotor (Beckman Coulter), at 160.000 g for 2.5 h at 4°C in an optima L‐100K ultracentrifuge (Beckman Coulter). Upon centrifugation, fractions of 1 mL were collected, labelled F1 (top) to F12 (bottom) and stored at 4°C.

### 2.7 EV Mimetic Purification

Strep‐Tactin XT 4flow high capacity 50% resin was used for purification. 10X Wash buffer (WB, 1 M Tris/HCl, 1.5 M NaCl, 10 mM EDTA, pH 8) and 10X elution buffer (EB, 1 M Tris/HCl, 1.5 M NaCl, 10 mM EDTA, 500 mM biotin, pH 8) were prepared, diluted to 1X right before use in dH2O. WB and EB was supplemented with 0.2% BSA, 25 mM Trehalose and 1X Protease inhibitor cocktail (PIC, Roche). Wash buffer and elution buffer containing BSA, Trehalose and PIC supplements are abbreviated to WB^+^ and EB^+^, respectively. 200 µL resin was washed two times with 1 mL WB^+^, followed by centrifugation for 1 min at 500 g and subsequent aspiration of the supernatant. Then, resin was mixed with 800 µL F5, followed by addition of 89 µL 10X WB to adjust pH. Incubation was performed for 1 h at 4°C. Resin was centrifuged for 1 min at 500 g, supernatant was collected, and WB^+^ was added again to wash the resin. This procedure was followed five times to wash the resin. Finally, EB^+^ was incubated with resin for 30 min at 4°C. After centrifugation for 1 min at 500 g, supernatant was collected. A second and third elution step was performed without any additional incubation time. For Figure S2B, purification was performed with WB and EB.

### 2.8 SDS‐Page

Samples were mixed with 4× Sample Buffer (320.95 mM Tris‐HCl, 277 mM SDS, 120 µM Bromophenol Blue, 200 mM DTT, 40% glycerol, in H_2_O) to a final concentration of 1× sample buffer. Samples were subsequently heated at 95°C for 5 min in closed tubes, cooled to RT and loaded onto a 4%–12% Bis‐Tris polyacrylamide gel (Thermo Fisher Scientific). Gel electrophoresis was performed at 165 V for 35 min in MES SDS buffer (Invitrogen). A Precision Plus Protein ladder (BioRad Laboratories) was used to determine molecular weight.

### 2.9 Silver Staining

Upon SDS‐Page, gel was rinsed with dH_2_O. Then, gel was fixed using freshly prepared 40% v/v ethanol, 10% v/v acetic acid in MQ, for 30 min at RT. Gel was then incubated using 30% v/v ethanol, 0.5 M sodium acetate, 25 mM sodium thiosulphate, 1% v/v Glutaraldehyde in MQ, for 30 min at RT. Glutaraldehyde was added just before use. Then, gel was rinsed three times for 10 min using MQ. Subsequently, a freshly prepared staining solution, containing 5.9 mM silver nitrate and 0.009% v/v formaldehyde in MQ, was incubated for 40 min at RT. Gel was rinsed once more with MQ. Then, developing was performed by incubation with 236 mM sodium carbonate and 0.0045% v/v formaldehyde in MQ. Developing was stopped using 50 mM EDTA in MQ, pH8. Gel was imaged at 700 nm using the Odyssey M Imager (LI‐COR Biosciences).

### 2.10 Western Blot

Upon SDS‐Page, proteins were transferred in blot buffer (25 mM Tris and 192 mM Glycin, ethanol 20% v/v in dH_2_O) to a PVDF membrane, that was pre‐activated for 1 min in methanol. Transfer was performed at 125 V for 65 min. Membrane was subsequently blocked using 50% v/v Odyssey Blocking Buffer (LI‐COR Biosciences) in Tris buffered saline (TBS). All immuno‐labellings were performed with 25% v/v Odyssey Blocking Buffer in TBS containing 0.1% Tween 20 (TBS‐T) and analysed using the Odyssey M Imager (LI‐COR Biosciences). Primary antibodies were used for 1h‐o/n and included Mouse Anti‐Strep (IBA, #2‐1517‐001, 1:1000), Rabbit Anti‐CD47 (Thermo Fisher Scientific, #PA5116827, 1:1000), Mouse Anti‐CD39 (ENTPD1) (Thermo Fisher Scientific, #CF804402, 1:500). Secondary antibodes were used for 30–60 min in the dark and included Alexa Fluor 680‐conjugated antimouse antibody (Thermo Fisher Scientific, A#21057, 1:7500) and Alexa Fluor 680‐conjugated antirabbit antibodies (Thermo Fisher Scientific, #A‐21076‐#A21076, 1:7500).

### 2.11 Immuno‐Electron Microscopy

Copper formvar grid were carbon‐coated using the BOC Edwards Auto 306 vacuum carbon sputter machine (Edwards Vacuum) or the EM ACE600 Sputter Coater (Leica Microsystems). Then, the coated grids were incubated with a 15–25 µL droplet of sample for 5–10 min. Subsequently, grids were washed three times with HBS. Then, for labelling performed with saponin, grids were incubated with 0.1% saponin in PBS for 5–10 min. Grids were washed three times with HBS. All grids were blocked for ≥10 min with 1% BSA in HBS (HBSA). Grids were washed three times with HBS and incubated with bridging antibody in HBSA for ≥10. Bridging was only needed for mouse primary antibody, as the Protein A Gold nanoparticles used directly bind rabbit primary antibodies. Upon bridging antibody incubation, grids were washed three times with HBS and incubated for 5–10 min with Protein A Gold nanoparticles of 15 nm diluted in HBSA. Grids were washed three times with HBS and fixed for 5 min with 1% glutaraldehyde in HBS. Grids were then washed three times with SuperQ. Grids were incubated for 10 min with uranyl acetate (pH 7) and consecutively for 10 min with uranyl acetate methyl cellulose (pH 4). Finally, the grids were looped out from the solution, by blotting on filter paper, and air‐dried. Grids that were not labelled with gold nanoparticles were incubated with sample, washed and directly fixed using glutaraldehyde. Then, grids were treated as described above. The grids were imaged using a Tecnai12 electron microscope (FEI Microscopy Solutions) at 80 kV. Antibodies used were diluted to 5 µg/mL in HBSA, except the bridge antibody, which was used at 1 µg/mL. Antibodies used were Mouse Anti‐Strep (IBA, #2‐1517‐001), Rabbit Anti‐FLAG (Merck KGaA, #F7425), Rabbit anti mouse bridge antibody (Rockland, #610‐4120), Mouse isotype IgG (BioLegend, #401402), Rabbit isotype IgG (Thermo Fisher Scientific, 10610274). Quantitative analysis of immuno‐EM images was performed by analysing images at 30.000× magnification, and individually counting liposomes and EV mimetics. For the quantification presented in Figure S2, three individual images were analysed per biological replicate, whereas for Figure S4, four individual images were analysed. Large aggregated particles were excluded from particle quantifications.

### 2.12 Nano Flow Cytometry

Mix, F5 and eluate samples were prepared as described above. Samples were diluted to a concentration of 1E10 particles/mL based on normalization of cy5 lipid fluorescence. Labelling was performed in 20 µL HBS containing 0.2% BSA, 25 mM Trehalose and 1X protease inhibitors. 1 µL of 100 µg/mL primary antibody was added to the sample and incubated overnight at 4°C. Then, 1 µL of 1 mg/mL AF488 conjugated secondary antibody was added and incubated overnight at 4°C. Samples were diluted 200 times in 0.02 µm filtered PBS right before analysis using a Nano Flow Cytometer (CytoFLEX nano, Beckman). Antibodies used were Mouse Anti‐Strep (IBA, #2‐1517‐001), Mouse Control IgG (Southern Biotech, 0102‐01), Goat Anti‐Mouse AF488 secondary (Thermo Fisher, A38175).

### 2.13 Proteinase K Protection Assay

For proteinase K protection assay, F5 was collected upon DGU and incubated with a final concentration of 50 mM proteinase K (Invitrogen) in presence or absence of 1% sodium dodecyl sulphate (SDS, Merck). Incubation was performed at 37°C for 1 h. After incubation, proteinase K activity was heat‐inactivated by incubation at 95°C for 10 min.

### 2.14 Nanoparticle Tracking Analysis

The concentration and size distribution of liposomes was measured using a Nanosight S500 nanoparticle analyser (Malvern Instruments) equipped with a 405 nm laser. Samples were diluted in HBS to a concentration that yielded 40–100 particles per frame. Videos were acquired with a camera level of 16 and processed using detection threshold of level 7. Five consecutive videos of 10s were recorded for each sample. These videos were analysed using NTA software version 3.4.

### 2.15 Cell Culture

MDA‐MB‐231 cells were cultured in Dulbecco's Modified Eagle Medium (DMEM) with L‐Glutamine (Gibco) supplemented with 10% foetal bovine serum (FBS), 100 U/mL penicillin and 100 µg/mL streptomycin (Gibco). Cells were routinely tested for mycoplasma contamination (Mycoplasma PCR Detection Kit, abm G238).

### 2.16 N‐Cadherin EV Mimetic Uptake Assay

Two cell‐free protein synthesis reactions were prepared: one with a plasmid for N‐Cadherin, and one with an N‐Cadherin mutant plasmid. For both mixes, DOPC/18:0Cy5PE (99.5/0.5) liposomes were used at 4.5 mM final concentration. Upon DGU, F5 containing EV mimetics was collected, and normalized based on fluorescence, by measuring fluorescence using the SpectraMax ID3 (Ex. 640, Em. 680). Using a refractometer, the concentration of sucrose in F5 was determined. Crude liposomes were then diluted in the same buffer, with an equal concentration of sucrose, and used as additional control.

MDA‐MB‐231 cells were washed, trypsinized, counted and seeded at 20.000 cells/well in a 96‐well plate in 100 µL medium on Day 1. On Day 2, 50 µL N‐Cadherin EV mimetics, N‐Cadherin mutant EV mimetics, crude liposomes, or sucrose buffer were added to the 100 µL culture medium in triplo. The final concentration of liposomes corresponded to 8.6 µM. Cells were placed back in the incubator upon addition. After 4 h, cells were washed with 100 µL PBS. Then, 50 µL Trypsin was added and incubated for 5 min at 37°C. Trypsin was then deactivated using 100 µL culture medium. Cells were transferred to an untreated V‐bottom 96 well plate, and centrifuged for 3 min at 500 g. then, supernatant was discarded, and cell pellets were resuspended in 250 µL PBS containing 1% FBS. Cells were transferred to an F‐bottom 96‐well plate, and measured using the BD FACS Canto II (BD Biosciences). APC‐A was measured to quantify uptake.

### 2.17 Fluorescence Microscopy

For fluorescence microscopy images, a glass 96‐well black culture microplate was used (Greiner, cat#655090). Prior to cell seeding, wells were coated with 0.01% Poly‐L‐Lysine for 20 min at RT, washed with sterile tissue culture grade water, and allowed to dry for 2 h before introducing cells and medium. 10,000 cells/well were seeded. On Day 1, liposomes were added at a final concentration of 36.4 µM. After 4 h, cells were washed three times with PBS, and fixed using 4% PFA in PBS for 15 min at RT. Cells were then washed three times with PBS, and blocked with 1% BSA and 0.15% glycin in PBS for 30 min at RT. Cells were again washed three times with PBS, and then treated with 1 ug/mL DAPI staining, and 1:1000 AF488 phalloidin staining (Thermo Fisher, A12379) in 1% BSA in PBS for 1 h at RT. Cells were finally washed three times with PBS and imaged using a Zeiss Axio Observer Z1 equipped with L Zeiss Fluar 40×/1.3 oil objective, Colibri LEDs (365, 470 and 625 nm) in combination with Zeiss filtersets 01, 10 and 50, respectively. Images were made using ZEN 2 software (Blue edition) including zstack module. Zstacks were made for all channels upon which a fast iterative deconvolution was performed using ZEN 2 software. Finally, an appropriate in focus stack was chosen.

### 2.18 Cell Viability Assay

A PURExpress reaction was prepared as described above, supplemented with DOPC liposomes containing 0.5% 18:0Cy5PE and paclitaxel at a 2% paclitaxel to lipid molar ratio, and a plasmid encoding for N‐Cadherin. A control mixture was prepared without plasmid DNA. Samples were subjected to DGU as described above.

MDA‐MB‐231 cells were seeded at a density of 5000 cells/well in a 96‐well plate. On Day 1, liposomes were added at a final lipid concentration of 22.4 µM. Upon incubation for 4 h at 37°C, cell medium was refreshed, and cells were allowed to expand for 3 days in 100 µL culture medium. Finally, 20 uL MTS assay reagent (CellTiter 96, Promega) was added to the wells, and, after incubation for 30 min, absorbance was assessed at 490 nm using a SpectraMax ID3.

### 2.19 CD39 EV Mimetic ATP Assay

To assess the ability of the CD39 EV mimetics to convert ATP to ADP and AMP, an ATP Assay kit was utilized (Cayman, #700410). CD39 EV mimetics were prepared and isolated using DGU. Residual ATP of the cell‐free protein synthesis reaction was then removed using Zebaspin desalting columns (MWCO 7 kDa, Invitrogen). This was performed three times in HBS containing 8% sucrose, 0.2% BSA and 1× PIC. Subsequently, F5 was incubated 1:1 with ATP solution from the kit to a final concentration of 52.8 nM, supplemented with CaCl_2_ to a final concentration of 5 mM CaCl_2_, and incubated for 20 min at 37°C. After incubation, the assay was conducted following manufacturer's instructions. Luminescence was measured at 560 nm using the SpectraMax iD3 reader.

Recombinant Human CD39 (rhCD39) was used as a positive control in the functionality assays. Deglycosylation of RhCD39 was performed using PNGaseF (NEB) following manufacturer's instructions. Deglycosylation was performed under both non‐denaturing and denaturing conditions and analysed using Coomassie Blue staining. Non‐denatured rhCD39 was used for functionality assays.

### 2.20 Statistical Analysis

Statistical analysis was performed using GraphPad Prism v9 (GraphPad Software).

## 3 Results

### 3.1 Successful Production of EV Mimetics

We first aimed to produce EV mimetics by incorporating full‐length membrane proteins into liposomes. We utilized the PURExpress coupled in vitro transcription and translation system for membrane protein synthesis (Tuckey et al. 2014). This cell‐free protein expression system enables co‐translational insertion of full‐length membrane proteins if the reaction is supplemented with exogenous lipid bilayers, such as liposomes (Lu et al. 2018).

As a model protein for optimizing conditions for preparation of EV mimetics, we selected Cluster of Differentiation 47 (CD47), a membrane protein containing five transmembrane domains (van Duijn et al. 2022). CD47 is known to be abundant on EVs from a wide variety of cell sources, such as mesenchymal stem cells and breast tumour cells (Xu et al. 2023; Kugeratski et al. 2021). CD47 plays a key role in immune evasion by interaction with its ligand, signal‐regulatory protein (SIRP) α (van Duijn et al. 2022). This interaction triggers a “don't eat me” signal, inhibiting phagocytosis and extending the circulation time of EVs in the bloodstream.

To produce EV mimetics, liposomes were mixed with the in vitro cell‐free protein synthesis components supplemented with disulfide bond enhancers, RNase inhibitor and a plasmid DNA template encoding for the protein of interest (Figure 1A). A construct encoding for CD47 and a construct encoding for Twin‐Strep‐tag‐CD47 – containing an N‐terminal purification tag—were tested first. Following incubation, sucrose density gradient ultracentrifugation (DGU) was performed to isolate EV mimetics (Figure 1A).

To allow tracking of liposome yield at different steps of the production process, we incorporated a fluorescent lipid, 18:0Cy5PE, in the liposomal formulation. DOPC/18:0Cy5PE (99.8/0.2) liposomes were prepared using thin film hydration, and extruded through 100 nm polycarbonate membranes. Liposomes were characterized by Nanoparticle Tracking Analysis (NTA), which revealed a mode size of ∼120 nm (Figure 1B).

We then isolated liposomes using DGU. Following DGU, 18:0Cy5PE lipid in the liposomal formulation was quantified in all gradient fractions, indicating that liposomes floated to fraction 5 (F5) of the gradient, representing their equilibrium buoyant density (Figure 1C). Liposomes incubated with a plasmid for CD47 or for Twin‐Strep‐tag‐CD47 showed a similar lipid distribution. A western blot labelled for Strep‐tag indicated that Twin‐Strep‐tag‐CD47 protein ended up in F5 and F12, suggesting that while some residual protein remained at the bottom of the gradient, Twin‐Strep‐tag‐CD47 also floated with liposomes to F5 (Figure 1C). This confirms the successful incorporation of Twin‐Strep‐tag‐CD47 into liposomes, indicating the successful production of EV mimetics.

To verify purity of the EV mimetics upon DGU, a silver staining was performed. Bottom fractions contained a wide variety of proteins, likely attributed to residual protein of the cell‐free protein synthesis reaction. In contrast, F5 only showed a clear band around 25 kDa, corresponding to CD47 (Figure 1D). The presence of higher molecular weight bands in F5 may be attributed to multimerizations of the protein. This result indicates that CD47 EV mimetics could be isolated with high purity using DGU.

It has been reported previously that full‐length membrane proteins insert into lipid bilayers during translation (co‐translation) rather than after translation (post‐translation) (Lu et al. 2018; Moritani et al. 2010). To confirm that CD47 inserted in a co‐translational fashion, incubation of the cell‐free protein synthesis reaction was performed with liposomes added either before or after transcription and translation. Western blot revealed that CD47 was synthesized upon incubation of the cell‐free protein synthesis mix. Furthermore, CD47 was detected in F5 upon DGU when liposomes were added before (Figure 1E, Co/C), but not when added after transcription and translation (Figure 1E, Post/P). This highlights the co‐translational nature of membrane protein insertion events during the cell‐free protein synthesis reaction.

Taken together, these results show successful production of CD47 EV mimetics upon co‐incubation of a cell‐free protein synthesis reaction, liposomes and plasmid DNA.

### 3.2 Optimization of EV Mimetic Yield

We next aimed to optimize yield of Twin‐Strep‐tag‐CD47 EV mimetic synthesis. We first optimized plasmid concentration, as optimal yield may vary for each template used (Tuckey et al. 2014). We tested a range from 5, 10, 20 and 40 ng/µL. Although plasmid concentration did not affect total protein production (Figure 2A, Mix), CD47 incorporation in liposomes was found to be most efficient at a plasmid concentration of 10 ng/µL (Figure 2A, F5).

There is some evidence that tailoring parameters such as the reaction temperature could benefit efficiency of membrane protein synthesis, by improving protein folding and preventing aggregation (Tuckey et al. 2014; Colant et al. 2021). Additionally, cell‐free protein expression systems typically consist of isolated components derived from host cells, which can result in reduced thermostability compared to their native cellular environment. Therefore, a lower reaction temperature might enhance membrane protein yield. We tested overnight reaction temperatures of 20°C, 30°C and 37°C, and an additional condition of 37°C in which the reaction was incubated for only 4 h. We observed a higher expression of CD47 for 37°C than for other temperatures tested (Figure 2B). This was observed in both total yield (Figure 2B, Mix) and liposomal insertion (Figure 2B, F5). No differences were observed between 4 h and o/n incubation in terms of yield. We therefore decided to use 37°C as reaction temperature for further experiments.

The presence of liposomes during cell‐free protein synthesis is essential for proper co‐translational folding into liposomes. Additionally, the concentration of liposomes may dictate downstream yield and prevent aggregation (Lu et al. 2018). Therefore, we titrated liposome concentration during cell‐free protein synthesis to optimize membrane protein insertion. We tested several concentrations of DOPC liposomes added during cell‐free protein synthesis, and assessed the efficiency of CD47 incorporation into liposomes using western blot. With increasing lipid concentration, we observed a slight increase in total protein production (Figure 2C, Mix). However, higher liposome concentrations significantly enhanced CD47 levels upon DGU, suggesting more efficient incorporation into liposomes (Figure 2C, F5). To assess whether this increase in EV mimetic yield is reflected in a higher amount of protein per particle, we visualized EV mimetics using immuno‐electron microscopy (immuno‐EM) (Figure 2D). CD47 in EV mimetics was observed by Strep‐tag antibody labelling, followed by labelling with 15 nm protein A gold nanoparticles. As observed by immuno‐EM, low concentrations of lipid (0.1–1 mM) yielded aggregated EV mimetics, possibly due to an excess of uninserted protein. Higher concentrations of lipid, that is, 5 and 10 mM, showed reduced EV mimetic aggregation. We observed that at a lipid concentration of 5 mM, protein aggregation was low. Additionally, a relatively high protein content was observed upon DGU using western blot. Immuno‐EM confirmed a relatively high labelling density per particle. Consequently, subsequent experiments were conducted using 5 mM total lipid concentration.

These results show that by optimizing reaction conditions, including plasmid concentration, reaction temperature and liposome concentration, EV mimetic production efficiency can be enhanced.

### 3.3 Successful Purification of EV Mimetics

**FIGURE 3:**
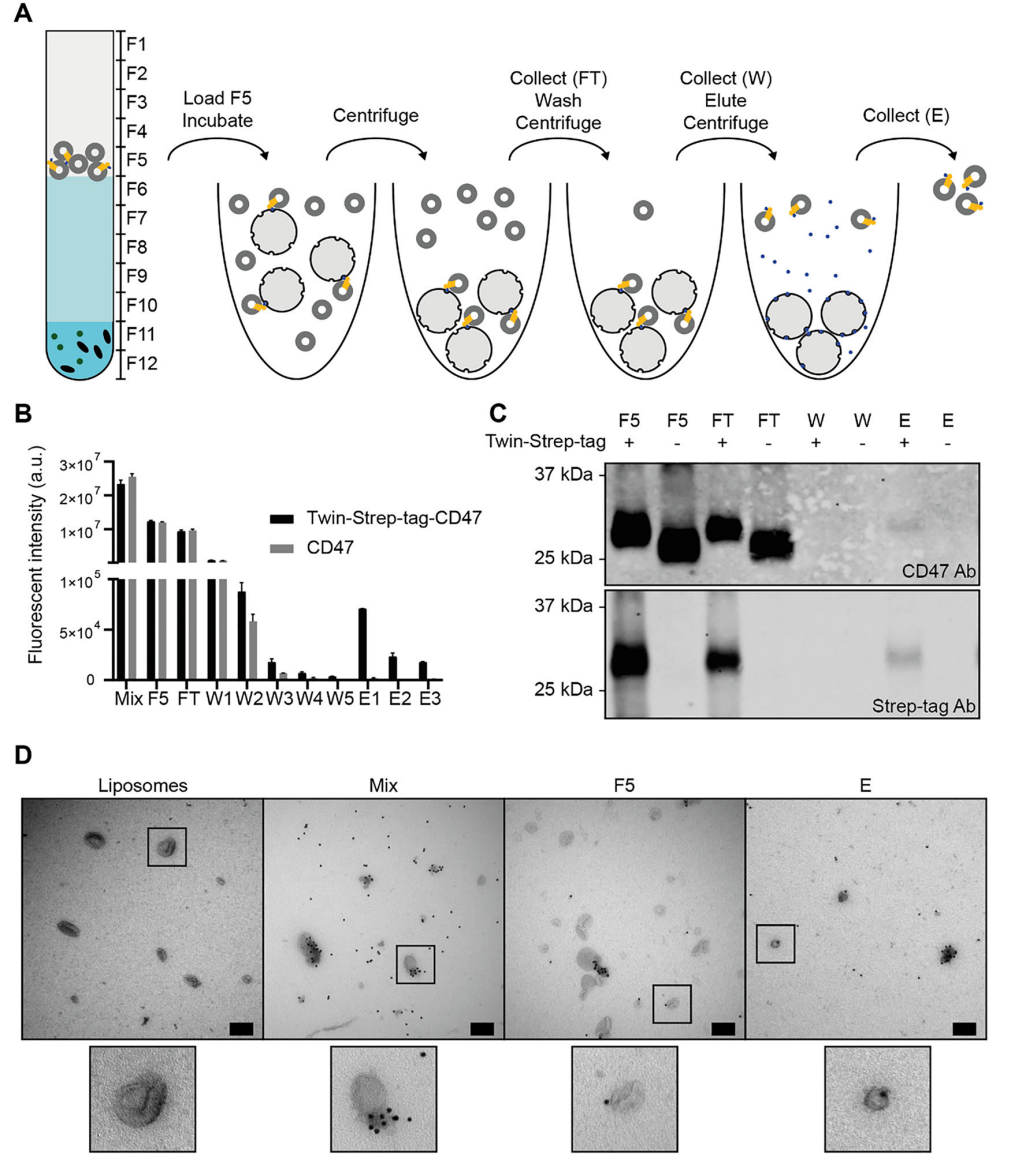
Strep‐tag affinity purification allows successful purification of EV mimetics. (A) Schematic overview of the procedure. Upon sucrose density gradient ultracentrifugation (DGU), Fraction 5 (F5) containing EV mimetics was incubated with Strep‐Tactin XT resin, allowing binding via Twin‐Strep‐tag, which is N‐terminally fused to CD47. Subsequently, the resin was pelleted by centrifugation, and a flow‐through (FT) sample was collected. Then, crude liposomes were washed and collected as wash (W) fractions. Upon repeated washings, Twin‐Strep‐tag‐CD47 EV mimetics were eluted using an excess of biotin, and collected as eluate (E). (B) Quantification of fluorescent liposomes collected at various stages of the purification process. Mean ± SD values are displayed. (C) CD47 and Strep‐tag labelled western blots showing samples taken at various stages of the purification process. (D) Immuno‐electron microscopy images of Strep‐tag labelled crude liposomes and Twin‐Strep‐tag‐CD47 EV mimetics at various stages of the purification process. Scale bar: 200 nm.

Following production and DGU of EV mimetics, a mixture of EV mimetics and crude liposomes is obtained. We thus next aimed to purify EV mimetics from crude liposomes. We first produced CD47 EV mimetics as well as Twin‐Strep‐tag‐CD47 EV mimetics. Then, to purify EV mimetics, we incubated F5 with Strep‐Tactin XT resin. After incubation, the resin was pelleted by centrifugation and washed. EV mimetics were then eluted using an excess of biotin (Figure 3A).

Upon quantification of fluorescent lipid at various stages of purification, we could observe liposomes present in the eluate of Twin‐Strep‐tag‐CD47 EV mimetics, but not of CD47 EV mimetics, indicating successful purification only in presence of Twin‐Strep‐tag (Figure 3B). The results indicate a fluorescent intensity of ∼7.5E4 a.u. in the eluate and a fluorescent intensity of ∼2E7 a.u. before performing DGU and subsequent affinity purification. This results in a final yield of about 0.25%. To confirm that purification yielded liposomes containing CD47, we analysed all samples upon purification using western blot analysis for expression of CD47 and Strep‐tag (Figure 3C). The CD47 labelled western blot revealed that CD47 was not detected in the eluate of CD47 EV mimetics. In contrast, CD47 was detected in the eluate of Twin‐Strep‐tag‐CD47 EV mimetics. The Strep‐tag labelled western blot indicated presence of CD47 in the eluate, further confirming successful purification of Twin‐Strep‐tag‐CD47 EV mimetics. These results confirm that purification of Twin‐Strep‐tag CD47 EV mimetics was successful.

To assess enrichment of Twin‐Strep‐tag‐CD47 EV mimetics upon purification, immuno‐EM was performed (Figure 3D). No gold labelling was detected on crude liposomes (Figure 3D, Liposomes). Additionally, isotype‐labelled Twin‐Strep‐tag‐CD47 EV mimetics showed no gold labelling (Figure S1). We observed extensive labelling upon cell‐free protein synthesis (Figure 3D, Mix). In addition to labelling on the liposomes, gold nanoparticles were observed throughout the image, likely representing unincorporated membrane proteins. Additionally, some crude liposomes without gold labelling were observed. Upon DGU, although some crude liposomes remained, most unincorporated membrane proteins were removed, indicating successful isolation of liposomes from unincorporated membrane proteins (Figure 3D, F5). After purification, only Twin‐Strep‐tag‐CD47 EV mimetics were observed, with no crude liposomes or excess unincorporated proteins present (Figure 3D, E). To quantify purity, we then assessed the total number of EV mimetics and plain liposomes (without CD47) in three individual images for three biologically independent experiments. Although the Mix and F5 samples contained between 40% and 60% EV mimetics, the eluate contained ∼75% EV mimetics, confirming successful enrichment using Twin‐Strep‐tag purification (Figure S2). Additionally, to further confirm enrichment of CD47 EV mimetics in the eluate, we performed single particle nano flow cytometry. Flow cytometric analysis revealed successful enrichment of EV mimetics in the eluate, as indicated by a higher percentage of CD47‐positive liposomes (61.6% for eluate vs. 40.9% for F5 and 48.6% for Mix) (Figure S3). These findings confirm the successful purification of EV mimetics.

Altogether, these results demonstrate successful purification of EV mimetics from crude liposomes.

### 3.4 Preservation of Membrane Protein Topology in EV Mimetics

Previously, it has been shown that translocon‐independent cell‐free protein expression systems can generate membrane proteins with inverted topology, when combined with lipid bilayers like those of liposomes (Pellowe and Booth 2020; Harris et al. 2017). These findings underscore the importance of evaluating membrane protein topology following EV mimetic synthesis. Therefore, we aimed to determine the topology of membrane proteins in EV mimetics.

**FIGURE 4:**
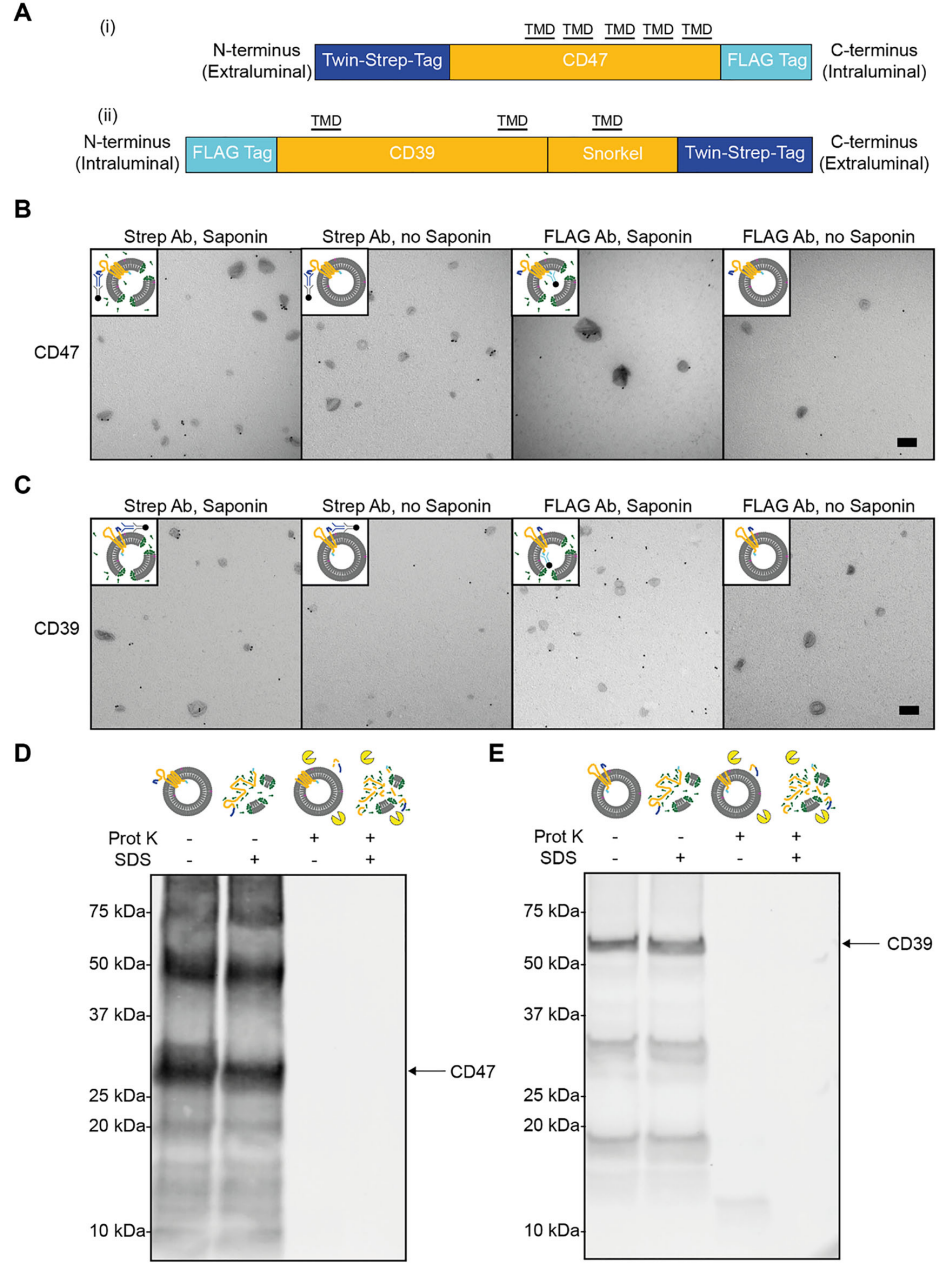
Membrane protein topology is preserved in EV mimetics. (A) Schematic overview of the protein constructs used. (i): CD47, (ii): CD39. TMD: Transmembrane domain. (B) Immuno‐electron microscopy images of CD47 EV mimetics, containing an extraluminal N‐terminal Twin‐Strep‐tag and an intraluminal C‐terminal FLAG‐tag. As indicated by the schematics, CD47 EV mimetics were labelled for Strep‐tag or FLAG‐tag in the absence and presence of saponin. Scale bar: 200 nm. (C) Immuno‐electron microscopy images of CD39 EV mimetics, containing an intraluminal N‐terminal Twin‐Strep‐tag and an extraluminal C‐terminal FLAG‐tag. As indicated by the schematics, CD39 EV mimetics were labelled for Strep‐tag or FLAG‐tag in the absence and presence of saponin. Scale bar: 200 nm. (D) Western blot labelled for Strep‐tag showing a proteinase K protection assay for CD47 EV mimetics, both in presence and in absence of SDS. (E) Western blot labelled for Strep‐tag showing a proteinase K protection assay for CD39 EV mimetics, both in presence and in absence of SDS.

To evaluate membrane protein topology, a CD47 protein construct with a tag on the N‐terminus and C‐terminus was designed, allowing labelling of both termini of the protein. The N‐terminus, which is extraluminal in EVs, was fused to a Twin‐Strep‐tag sequence. The C‐terminus, which is intraluminal in EVs, was fused to a FLAG‐tag sequence (Figure 4A, i).

In addition to CD47, we selected Cluster of Differentiation 39 (CD39). CD39 is an ecto‐enzyme that, unlike CD47, necessitates an intraluminal orientation of its N‐terminus to maintain proper functionality (Dwyer et al. 2007; Grinthal and Guidotti 2006). The N‐terminus, which is intraluminal for CD39 in EVs, was labelled with a FLAG‐tag sequence. Given that CD39 contains an intraluminal C‐terminus in EVs, we incorporated a modified SnorkelTag sequence at the C‐terminus, which introduces an additional transmembrane domain followed by a C‐terminal Twin‐Strep‐tag sequence (Figure 4A, ii) (Brown et al. 2013). This strategy allows purification without altering the functional extracellular epitope of CD39.

We then produced EV mimetics and analysed membrane protein topology using immuno‐EM and a proteinase K protection assay. First, we labelled EV mimetics using either Strep‐tag antibody or a FLAG‐tag antibody, in presence and absence of saponin to permeabilize liposomes, allowing labelling of epitopes located in the lumen of the EV mimetics. Samples were then visualized using immuno‐EM. For CD47 EV mimetics, we observed labelling of Strep‐tag, regardless of the presence of saponin. However, FLAG‐tag labelling was only observed in the presence of saponin, indicating saponin is required to expose the FLAG epitope (Figures 4B and S4A). These findings suggest that CD47 EV mimetics contain membrane proteins that predominantly adopt their native topology. For CD39, similar to CD47 EV mimetics, we observe labelling of Strep‐tag regardless of the presence of saponin. Additionally, similar to CD47 EV mimetics, we observe FLAG‐tag labelling only in the presence of saponin, indicating that CD39 adopts a favourable membrane protein topology (Figures 4C and S4B). This is particularly interesting, as this indicates that two proteins, with either an intraluminal or extraluminal N‐terminus, can spontaneously insert in the lipid bilayer of liposomes in their native membrane protein topology. Using isotype controls, we confirmed that no gold labelling is visualized without a specific Strep‐tag or FLAG‐tag antibody (Figure S1). Taken together, these results are consistent with a predominantly correct membrane protein topology for both CD47 and CD39 EV mimetics.

Next, to further confirm native membrane protein topology, we performed a proteinase K protection assay. EV mimetics were incubated with proteinase K, either in the presence or absence of SDS, which disintegrates the liposomes. Western Blot revealed that both CD47 and CD39 EV mimetics lost their Twin‐Strep‐tag epitope upon incubation with proteinase K, regardless of the SDS presence (Figure 4D, E). This indicates that indeed the epitope of Twin‐Strep‐tag is located on the extraluminal side of the EV mimetics, further solidifying the hypothesis that the membrane proteins are correctly folded.

These findings confirm correct protein topology of CD47 and CD39 EV mimetics in the lipid bilayer of liposomes.

### 3.5 Preservation of Membrane Protein Functionality in EV Mimetics

To examine membrane protein functionality of EV mimetics, we first selected CD39. CD39 functions as an ecto‐enzyme that converts adenosine triphosphate (ATP) and adenosine diphosphate (ADP) to cyclic adenosine monophosphate (cAMP) (Dwyer et al. 2007l Fuentes and Palomo 2015). Consequently, ATP reduction serves as an indicator of CD39 enzymatic activity. CD39 has been detected on the surface of EVs from tumour cells and immune cells, including T cells (Schuler et al. 2014; Ludwig et al. 2020; Clayton et al. 2011). Therefore, we aimed to synthesize and purify CD39 EV mimetics and evaluate enzymatic activity of the EV mimetics formed. Even though synthesis and purification of CD39 EV mimetics was successful, enzymatic activity could not be observed. Alternatively, recombinant human CD39 readily reduced ATP, and this enzymatic activity was lost upon deglycosylation of this protein, indicating importance of N‐glycosylations for functionality (Figure S5). The current strategy we adopted for EV mimetic synthesis lacks the ability to glycosylate proteins (Hunt et al. 2025). Therefore, we aimed to explore functionality of a different membrane protein.

**FIGURE 5:**
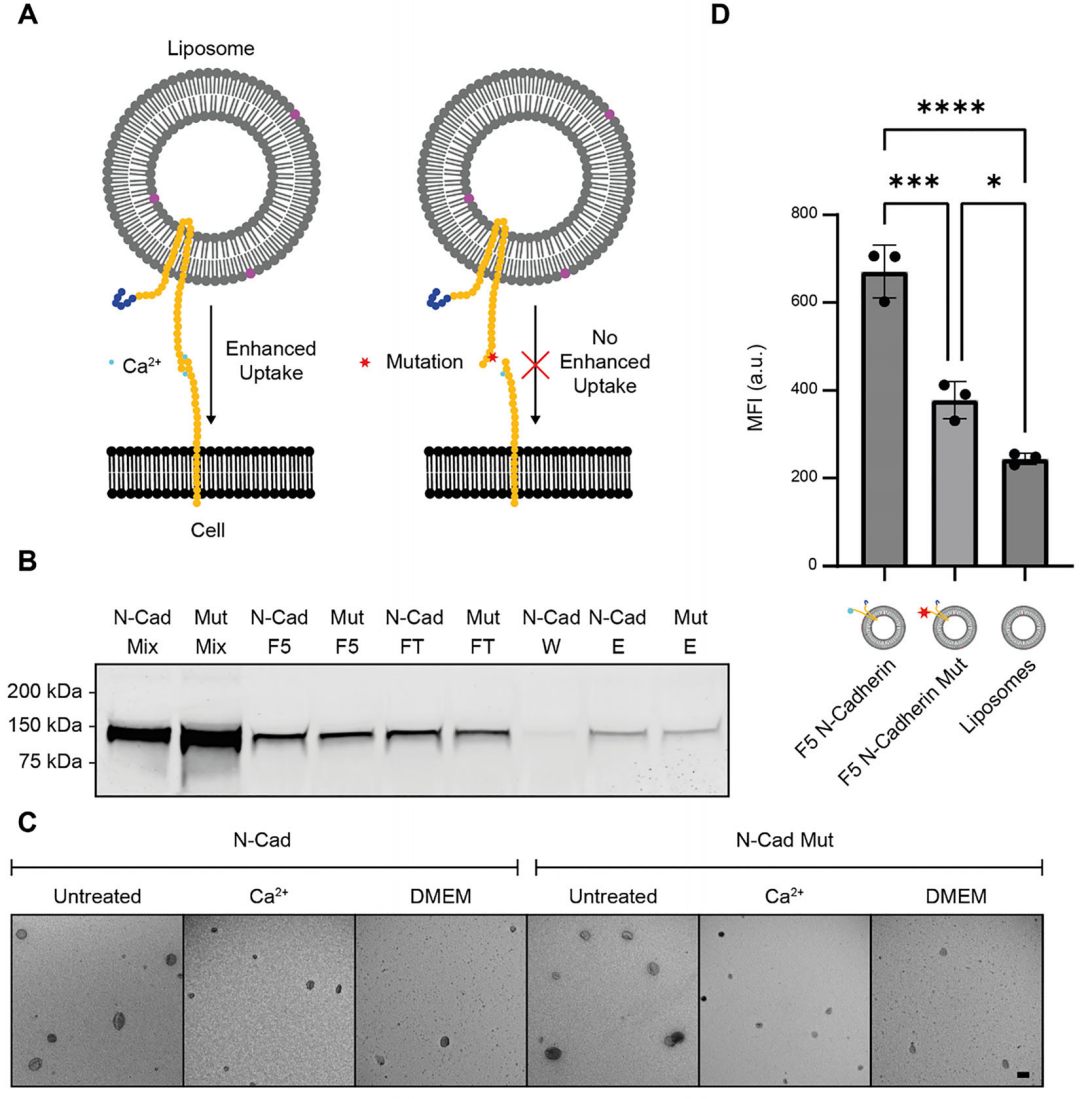
Membrane protein functionality is preserved in EV mimetics. (A) Schematic overview of EV mimetic uptake by MDA‐MB‐231 cells. EV mimetics containing either N‐Cadherin or a mutant N‐Cadherin were added to N‐Cadherin expressing MDA‐MB‐231 cells. Next, uptake was assessed using fluorescent lipids in the N‐Cadherin EV mimetics. (B) Strep‐tag labelled western blot showing samples taken at various stages of the purification process of N‐Cadherin EV mimetics and N‐Cadherin mutant EV mimetics. (C) Electron microscopy images of N‐Cadherin and N‐Cadherin mutant EV mimetics upon density gradient ultracentrifugation (DGU), either left untreated, or treated with Ca^2+^ or DMEM containing Ca^2+^ upon DGU. Scale bar: 200 nm. (D) EV mimetic uptake by MDA‐MB‐231 cells after 4 h incubation, analysed using flow‐cytometry. One‐way ANOVA Tukey's multiple comparison test was used for statistical analysis, *p < 0.05, ****p < 0.00001, individual values ± SD are displayed.

We selected Neural Cadherin (N‐Cadherin), also known as Cadherin‐2, for functional testing (Radice 2013). N‐Cadherin is a transmembrane cell‐cell adhesion molecule, containing a single transmembrane domain. In the presence of Ca^2+^, N‐Cadherin is known to undergo homophilic binding. Beyond its role in adhesion, it has an import role in cell signalling and maintaining tissue morphology. N‐cadherin is known to be overexpressed on the cell surface of cells that undergo tumour progression, and is a key player in endothelial‐to‐mesenchymal (EMT) transitioning (Cao et al. 2019). Therefore, N‐Cadherin EV mimetics could possibly be used for targeted delivery to tumorigenic cells (Liu et al. 2024). Integration of N‐Cadherin in liposomes has been shown before, resulting in enhanced uptake in human glioblastoma cells (Kamiya et al. 2011). Importantly, it has been described that N‐Cadherin does not lose its binding properties in absence of glycosylations, rendering it an interesting choice for functionality studies (Guo et al. 2009; Langer et al. 2012). The functionality of N‐Cadherin EV mimetics was tested by evaluating their uptake by MDA‐MB‐231 cells that are known to express N‐Cadherin (Naderi et al. 2022; Huang et al. 2020). A well described aspartic acid to alanine point mutation of N‐Cadherin (D134A), prevents binding of Ca^2+^ between two extracellular domains of N‐Cadherin (Harrison et al. 2005). This mutation impedes homophilic binding and served as a negative control (Figure 5A).

First, we prepared N‐Cadherin EV mimetics and N‐Cadherin mutant EV mimetics using fluorescent liposomes (DOPC/18:0Cy5PE (99.5/0.5)). Next, we performed a purification of N‐Cadherin EV mimetics, and evaluated N‐Cadherin content by western blot. Western blot analysis revealed comparable levels of N‐Cadherin in both N‐Cadherin and N‐Cadherin mutant EV mimetics, both upon DGU and upon affinity purification (Figure 5B). This indicates that both N‐Cadherin EV mimetics and N‐Cadherin mutant EV mimetics were successfully produced and purified at similar concentrations.

As N‐Cadherin is a homophilic adhesion molecule, we examined EV mimetic aggregation upon DGU. To this end, EV mimetics were imaged using EM upon DGU. Samples were either left untreated or pre‐treated with CaCl_2_ (Ca^2+^) or full medium (DMEM), at a concentration of 1.2 mM CaCl_2_, which is equal to the final concentration used during incubation with MDA‐MB‐231 cells. None of the samples showed any sign of aggregation, indicating that the presence of N‐cadherin on EV mimetics did not induce their aggregation (Figure 5C).

EV mimetics were then incubated with MDA‐MB‐231 cells for 4 h. We first confirmed that particles are taken up by MDA‐MB‐231 cells by analysing z‐stack fluorescent microscopy images (Figure S8). We then analysed uptake using flow cytometry as determined by the amount of fluorescent signal in the cells. N‐Cadherin EV mimetics showed significantly higher uptake than N‐Cadherin mutant EV mimetics and crude liposomes, indicating that N‐Cadherin in EV mimetics promotes uptake by MDA‐MB‐231 cells (Figure 5D). Finally, we tested the drug delivery potential of the EV mimetics formed. To this end, we formulated liposomes loaded with paclitaxel, and tested cell viability upon treatment of MDA‐MB‐231 cells with liposomes or N‐Cadherin EV mimetics using an MTS assay. We observed lower viability for cells treated with N‐Cadherin EV mimetics, indicating enhanced functionality with respect to plain liposomes (Figure S7).

Taken together, these results indicate that the functionality of N‐Cadherin is preserved in EV mimetics.

### 3.6 Lipid Versatility and Dual Insertion of Membrane Proteins in EV Mimetics

**FIGURE 6:**
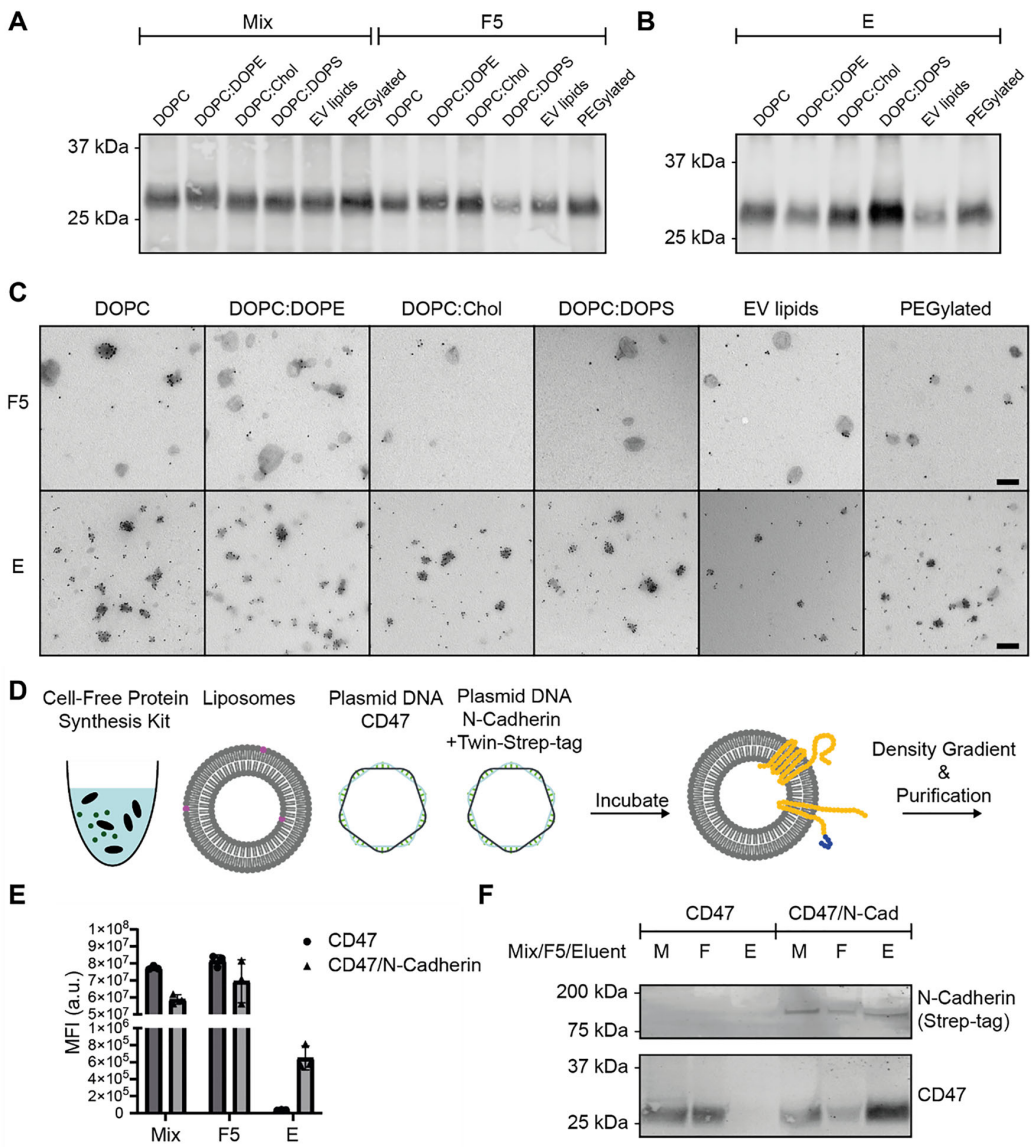
EV mimetics can be produced with a wide variety of liposomal formulations, and two different membrane proteins can be inserted in the same EV mimetic. Formulations are, from left to right: DOPC, DOPC/DOPE (3/1), DOPC/Chol (3/1), DOPC/DOPS (9/1), DOPC/EggSM/Chol/DOPS/DOPE (21/17.5/30/14/17.5), DOPC/DOPE/DSPE‐PEG‐2000 (73/25/2). (A) Strep‐tag labelled Westen blot of EV mimetics upon production (mix) and isolation after density gradient ultracentrifugation (DGU), labelled Fraction F5 (F5). (B) Strep‐tag labelled western blot of Twin‐Strep‐tag‐CD47 EV mimetics upon purification. (C) Strep‐tag labelled immuno‐electron microscopy images upon DGU and purification. Scale bar: 200 nm. (D) Schematic overview of experimental setup: Cell‐free protein synthesis is performed in presence of two different plasmids, encoding for CD47 devoid of a Twin‐Strep‐tag and N‐Cadherin containing a Twin‐Strep‐tag. Following synthesis, EV mimetics are purified using DGU and purification. (E) Quantification of fluorescent liposomes collected at various stages of the purification process. (F) Strep‐tag and CD47 labelled western blots of CD47 EV mimetics and CD47/N‐Cadherin EV mimetics collected at various stages of the purification process.

To explore the feasibility to tailor liposomal formulations for Twin‐Strep‐tag‐CD47 EV mimetic synthesis, we selected six distinct liposomal formulations: DOPC, DOPC/DOPE (3/1), DOPC/Chol (3/1), DOPC/DOPS (9/1), DOPC/EggSM/Chol/DOPS/DOPE (21/17.5/30/14/17.5) (“EV lipids”), DOPC/DOPE/DSPE‐PEG‐2000 (73/25/2) (“PEGylated”). In addition to DOPC, which served as a primary lipid component, other widely utilized lipids in drug delivery were incorporated based on their functional properties. Additionally, an EV mimetic lipid formulation was prepared, designed to closely resemble the lipid composition of natural EVs (Lu et al. 2019). Each formulation was co‐incubated with the cell‐free protein expression system at a final lipid concentration of 5 mM, followed by DGU and subsequent affinity purification using Twin‐Step‐Tag‐CD47. EV mimetic synthesis and purification efficiency was then analysed using western blot and immuno‐EM. Across all tested formulations, we observed presence of Twin‐Strep‐tag‐CD47 EV mimetics upon synthesis, following DGU, and upon purification (Figure 6A,B). This indicates successful production and purification for each formulation tested. Furthermore, Strep‐tag labelling density was increased upon purification of EV mimetics (Figure 6C). In addition, NTA revealed that all formulations yielded particles with comparable size distributions and concentrations at each step of the process (Figure S8). Altogether, these results show that Twin‐Strep‐tag‐CD47 EV mimetics can be produced using a wide variety of liposomal formulations.

Finally, we explored the feasibility of incorporation of two distinct membrane proteins into a single EV mimetic. To achieve this, we co‐incubated a plasmid encoding for N‐Cadherin with a Twin‐Strep‐tag sequence, and a plasmid encoding for CD47 devoid of a Twin‐Strep‐tag sequence (Figure 6D). This design ensured that only N‐Cadherin–containing EV mimetics were retained during purification, while CD47 EV mimetics were removed upon washing of StrepTactin XT resin. CD47 will thus only co‐elute with N‐Cadherin EV mimetics if it is present on the same EV mimetic. Based on prior optimization (Figure 2A), the final plasmid concentration was determined to be 10 ng/µL, and consequently, each plasmid was added at a final concentration of 5 ng/µL. Upon quantification of fluorescent liposomes upon purification, we found no signal in the eluate when only CD47 was expressed. However, when the plasmids were co‐incubated, fluorescent signal could be observed in the eluate (Figure 6E). Subsequent western blot analysis, labelled for CD47 and Strep‐tag, revealed that CD47 was detected in the eluate of the CD47/N‐Cadherin EV mimetics, confirming that CD47 co‐elutes with N‐Cadherin EV mimetics upon purification (Figure 6F). These findings demonstrate that co‐incubation of the two plasmids enables the expression of two distinct membrane proteins on the same EV mimetic.

In conclusion, these results demonstrate the flexibility of producing a wide variety of EV mimetics by modifying liposomal formulations, which can be tailored for specific applications. Furthermore, we show that two distinct membrane proteins can be inserted in the same EV mimetic, highlighting the potential development of EV mimetics comprising multiple membrane proteins. Overall, these findings underscore the versatility and broad applicability of the described methodology.

## 4 Discussion

Extracellular vesicles (EVs) are promising drug delivery candidates, with potential advantages over synthetic delivery systems, such as better biocompatibility and presence of natural targeting ligands. However, their low yield, inefficient cargo loading and heterogeneity make them challenging to translate for clinical purposes. Here, we introduce EV mimetics, which are liposomal carriers with integrated full‐length membrane proteins. By synthesizing EV mimetics in a controlled environment, we aim to alleviate issues related to using EVs as drug delivery systems. As model EV‐associated membrane proteins, we selected CD47, CD39 and N‐Cadherin. We co‐incubated a cell‐free protein synthesis extract with liposomes and a plasmid encoding for the membrane protein of interest. Then, we demonstrated successful synthesis of EV mimetics by western blot, silver staining and immuno‐EM.

Plasmid concentration, reaction temperature and lipid concentration were optimized to increase EV mimetic yield. Using western blot and immuno‐EM we showed that membrane protein synthesis could be enhanced by supplementation of higher concentrations of liposomes. This is in accordance with previously reported results regarding cell‐free protein synthesis. For example, it has been shown that nascent chains of membrane proteins can aggregate, causing ribosomal aggregation, which can be prevented by increasing presence of chaperoning lipid bilayers (Steinkühler et al. 2024). Other efforts have been made to increase membrane protein expression. For example, Ando et al. aimed to increase interactions between nascent chain and lipid bilayer by fusing the protein of interest to an N‐terminal his tag, and by adding nickel containing lipids to the liposomal formulation (Ando et al. 2018). This must be used with consideration, however, as increasing the interaction of the N‐terminus of the membrane protein of interest with the lipid bilayer could possibly cause conformational changes.

Following synthesis, we aimed to purify EV mimetics using DGU followed by affinity purification. The location of the Twin‐Strep‐tag sequence in the protein design will likely impact its downstream functionality and affinity binding capacity. For N‐Cadherin, it is described that the N‐terminus is essential to its homophilic binding capacity. Therefore, we decided to fuse the Twin‐Strep‐tag sequence to the C‐terminus. As the C‐terminus is intraluminal, we fused an additional transmembrane domain to N‐Cadherin, inspired by the snorkel sequence (Brown et al. 2013). For CD39, it is known that the functional epitope is the extraluminal part of the protein. As both the N‐terminus and the C‐terminus of the protein are intraluminal, we decided to fuse this protein to a snorkel sequence at the C‐terminus as well, followed by a C‐terminal Twin‐Strep‐tag. Careful consideration is needed to tailor each protein sequence, both by considering downstream functionality as well as binding capacity during affinity purification. A possible alternative could be to incorporate cleavable tags such as the Tobacco Etch Virus protease on the protein of interest (Shih et al. 2005). However, careful consideration is required when designing protein constructs, as modifications can potentially affect translation efficiency and protein structure.

Using fluorescent liposome quantification and western blot, we could observe successful purification of CD47, CD39 and N‐Cadherin EV mimetics. Despite successful purification of EV mimetics, a significant portion of our input material was not captured during affinity purification and was instead found in the flow‐through (Figure 3B,C). As subsequent topology assays revealed correct membrane protein orientation (Figure 4), we are unsure how to explain low binding efficacy. Potentially, presence of excipients during purification might complicate effective binding. However, we observed that the presence of excipients is essential for the stability of the EV mimetics, as omitting the excipients resulted in absence of CD47 EV mimetics in the eluate (Figure S9A). An alternative explanation for inefficient purification is that only a subset of the epitopes of Twin‐Strep‐tag are available for binding to the Strep‐Tactin XT resin. We therefore performed purifications using constructs of CD47 that contained a flexible or rigid amino acid linker fused in between Twin‐Strep‐tag and CD47, aiming to maximize tag availability. Unfortunately, this did not result in more CD47 EV mimetics in the eluate, indicating that it is unlikely that tag availability is impaired (Figure S9B). In future work, EV mimetic yield could possibly be further enhanced by alleviating issues related to binding efficiency, potentially by tailoring excipients, or the use of an alternative affinity purification tag.

Immuno‐electron microscopy and proteinase K protection assays revealed that both CD47 (extraluminal N‐terminus) and CD39 (intraluminal N‐terminus) EV mimetics show correct protein topology. Correct translocon‐unassisted membrane protein orientation upon cell‐free protein synthesis has been reported before. For example, Harris et al. have shown that membrane protein GlpG could be inserted in its correct orientation (Harris et al. 2017). They employed a similar strategy, using liposomes as chaperone rather than translocase components. In this work, as well as in other works published by the same group, a proposed mechanism for co‐translational folding unassisted by endogenous translocase components was presented (Pellowe and Booth 2020; Harris et al. 2017; Brady et al. 2022). The proposed models suggest that during translation, alpha helical structures are formed that heavily interact with the lipid bilayer of, for example, liposomes. Thereafter, these alpha helices spontaneously insert into the lipid bilayer due to favourable hydrophobicity interactions. We here showed that co‐incubation of liposomes during translation is required for proper protein insertion, which has also been observed by others (Lu et al. 2019; Moritani et al. 2010). This implies that membrane proteins, once fully translated in absence of a lipid bilayer, cannot effectively insert in the lipid bilayer. Interestingly, recent models regarding transmembrane domain insertion in the lipid bilayer in the presence of translocases suggest that some transmembrane domains of the emerging nascent chain insert in the lipid bilayer without direct assistance of the translocon (Cymer et al. 2015). This suggests that translocon‐unassisted transmembrane domain insertion is a naturally occurring effect rather than an entirely synthetic process.

We then aimed to assess functionality of the EV mimetics synthesized. As CD39 is an ecto‐enzyme that reduces ATP and ADP to AMP, we quantified ATP concentrations upon incubation with CD39 EV mimetics. We did not observe enzymatic activity of CD39 EV mimetic. Enzymatic activity was observed for recombinant human CD39, but not when recombinant human CD39 was deglycosylated, indicating that glycosylation is essential for proper CD39 functionality. The PURExpress cell‐free transcription and translation system utilized in this work lacks an endogenous glycosylation machinery (Yue et al. 2023). For many proteins, glycosylations are important if not essential to their function. CD39 is an example of a protein that heavily relies on glycosylations for proper enzymatic function (Wu et al. 2005). To improve upon current cell‐free protein expression systems, research has been done to produce glycosylated proteins in vitro. A common method is supplementation of cell‐free protein synthesis systems with microsomes, which are extracts from the endoplasmic reticulum containing oligosaccharide transferases (OSTs) essential for glycoprotein synthesis (Hershewe et al. 2020). However, microsomes are vesicle‐like structures in which proteins typically translocate for post‐translational modification, preventing their co‐translational insertion into liposomes. A possible alternative strategy is the addition of purified OSTs (Yue et al. 2023). However, OSTs contain multiple membrane‐linked subunits, limiting effective expression (Helenius and Aebi 2001). Additionally, the reliance on lipid bilayer insertion complicates downstream purification from EV mimetics. Recent advances explore OST‐independent glycosylation, such as using soluble N‐glycosyltransferases (NGTs) for simple protein glycosylations (Kightlinger et al. 2019). However, NGTs cannot yet replicate complex eukaryotic glycosylations (Hershewe et al. 2020; Kightlinger et al. 2019). An alternative strategy involves expressing membrane proteins in host cells, isolating them via membrane solubilization, and reconstituting them in lipid bilayers (Rigaud and Lévy 2003). This technique allows isolation of membrane proteins from mammalian cells, while preserving their glycosylations (Butler and Spearman 2014). However, isolation of membrane proteins requires detergents like SDS or urea, which can (partially) denature proteins and complicate refolding (Visudtiphole et al. 2005; Dornmair et al. 1990). Additionally, detergent residues need to be removed, and protein orientation must be carefully optimized to maintain functionality (Rigaud and Lévy 2003; Popot 2014). Given these limitations, we believe a bottom‐up approach is a more suitable strategy for drug delivery applications.

We next tested the functionality of N‐Cadherin EV mimetics, by assessing uptake in N‐Cadherin MDA‐MB‐231 cells. N‐Cadherin EV mimetics, but not point‐mutated N‐Cadherin EV mimetics, show enhanced uptake in MDA‐MB‐231 cells, indicating enhanced uptake mediated by homophilic binding of N‐Cadherin. Furthermore, we showed that treatment of MDA‐MB‐231 cells with paclitaxel‐loaded N‐Cadherin EV mimetics resulted in lower cell viability compared to treatment with plain paclitaxel‐loaded liposomes, indicating the potential of EV mimetics for drug delivery applications. These results show that EV mimetics retain membrane protein functionality upon insertion into the lipid bilayer.

By showing that a wide variety of liposomal formulations could be used to form EV mimetics, we highlight the broad applicability of the methodology presented. We selected several lipids that might be useful for downstream applications. We included DOPE, a fusogenic lipid, due to its potential to enhance cargo delivery in downstream applications (Cheng and Lee 2016). Cholesterol was selected for its role in improving membrane stability (Nsairat et al. 2022). DOPS, an anionic lipid, was selected due to its potential modulatory effects on protein folding and translation efficiency (Lu et al. 2018). A PEGylated lipid was also tested, as PEGylation is commonly used to enhance stealth properties of liposomes (Nsairat et al. 2022). Finally, to more closely resemble physicochemical properties of EVs, we selected an EV mimetic lipid formulation designed to closely resemble the lipid composition of natural EVs (Lu et al. 2019). We were able to show that liposomal formulation could be adapted to suit downstream applications. An interesting endeavour for future research may be addition of ionizable lipids to the lipid formulation. Selecting ionizable lipids for future formulations enables more efficient loading of nucleic acids in liposomes (Schlich et al. 2021). Caution is required when selecting cationic lipids for nucleic acid loading of EV mimetics, as they may cause aggregation with negatively charged ribosomes, as previously reported for cell‐free expression systems (Ando et al. 2020).

Additionally, we tested if two distinct membrane proteins could be inserted in the same EV mimetic. Using western blot, we could confirm the presence of both N‐Cadherin and CD47 on a single EV mimetic. These results highlight the potential for development of EV mimetics with several distinct membrane proteins and thus functionality.

Research employing a bottom‐up strategy similar to the one presented in this article has also been conducted. For example, Lu. et al. introduced a bottom‐up strategy to incorporate connexin 43 into liposomes using a cell‐free protein expression strategy (Lu et al. 2019). These so‐called exosome‐mimetic particles were then shown to have enhanced uptake in U87 cells with respect to conventional liposomes. Furthermore, a nucleic acid loading strategy was presented. As an alternative methodology, Staufer et al. present a strategy for bottom‐up synthesis of fully synthetic EV mimetics (Staufer et al. 2021). His‐tagged peptides were coupled to liposomes formulated with lipids containing a nickel head group. In vitro testing of the synthetic EVs shows that similar to natural EVs, synthetic EVs were capable of exerting wound healing properties. To the best of our knowledge, however, ours is the first reported work that employs a purification strategy to obtain EV mimetics that contain enriched full‐length membrane proteins.

As an alternative top‐down strategy for generating EV mimetics, hybrid EVs have been studied (Rodríguez and Vader 2022; Sun et al. 2023). Hybrid EVs are a class of nanoparticles that consist of EVs as well as a synthetic carrier such as a liposome, fused by, for example, extrusion, sonication, or simple co‐incubation. Hybrid EVs, just like EV mimetics presented in this study, have potential benefits such as enhanced cargo delivery and advantages in terms of biocompatibility, with the additional advantage that they comprise proteins that are produced by cell sources and thus post‐translationally modified. Furthermore, EVs from in vitro cell sources contain a larger variety of membrane proteins that could possibly further enhance its functionality. However, some limitations such as limited encapsulation efficiency, lack of standardization and lack of control over membrane protein orientation still need to be properly addressed (Rodríguez and Vader 2022; Chen et al. 2025).

Further research is required to advance the clinical development of EV mimetics as drug delivery systems. For instance, as discussed above, yield could be improved by optimizing the purification strategy. Moreover, storage stability should be systematically evaluated to assess long‐term functionality of the EV mimetics. Batch‐to‐batch variability in purification efficiency and yield also needs thorough evaluation. Loading of biological cargo such as RNA could be explored, potentially by incorporating ionizable lipids into the liposomal formulation. Lastly, assessment of *in vivo* biodistribution of the N‐Cadherin EV mimetics could provide valuable insights for further applications. Collectively, these efforts may support the translation of EV mimetics toward clinical applications.

In conclusion, we demonstrate successful synthesis and purification of CD47, CD39 and N‐Cadherin EV mimetics. This was achieved by co‐incubating liposomes with a cell‐free protein expression system and a plasmid encoding for the protein of interest. By tailoring plasmid concentration, reaction temperature and liposome concentration, we could optimize reaction conditions to increase CD47 EV mimetic yield. Topology assays showed that both CD47 (with an intraluminal N‐terminus) and CD39 (with an extraluminal N‐terminus) were inserted with correct membrane protein orientation into liposomes. Furthermore, we show that functionality of N‐Cadherin in EV mimetics is preserved. We demonstrate broad applicability of the methodology by showing successful CD47 EV mimetic synthesis and purification using a wide range of lipid formulations. Finally, we show that two distinct membrane proteins could be inserted in a single EV mimetic. This study provides a meaningful step toward addressing challenges in EV‐inspired drug delivery development.

## Conflicts of Interest

No, there is no conflict of interest.

## Supporting information




**Supplementary Materials**: jev270190‐sup‐0001‐SuppMat.docx

## Data Availability

The data that support the findings of this study are available from the corresponding author upon reasonable request.
