# Supplementary material for: Bottom‐Up Synthesis and Purification of Extracellular Vesicle Mimetics
Source: J Extracell Vesicles. 2025 Nov 18;14(11):e70190. doi: 10.1002/jev2.70190 (PMC12626166; doi:10.1002/jev2.70190)
Supplement: Supplementary file 1 — Supplementary Materials: jev270190‐sup‐0001‐SuppMat.docx [file JEV2-14-e70190-s001.docx]

# Supplementary Materials


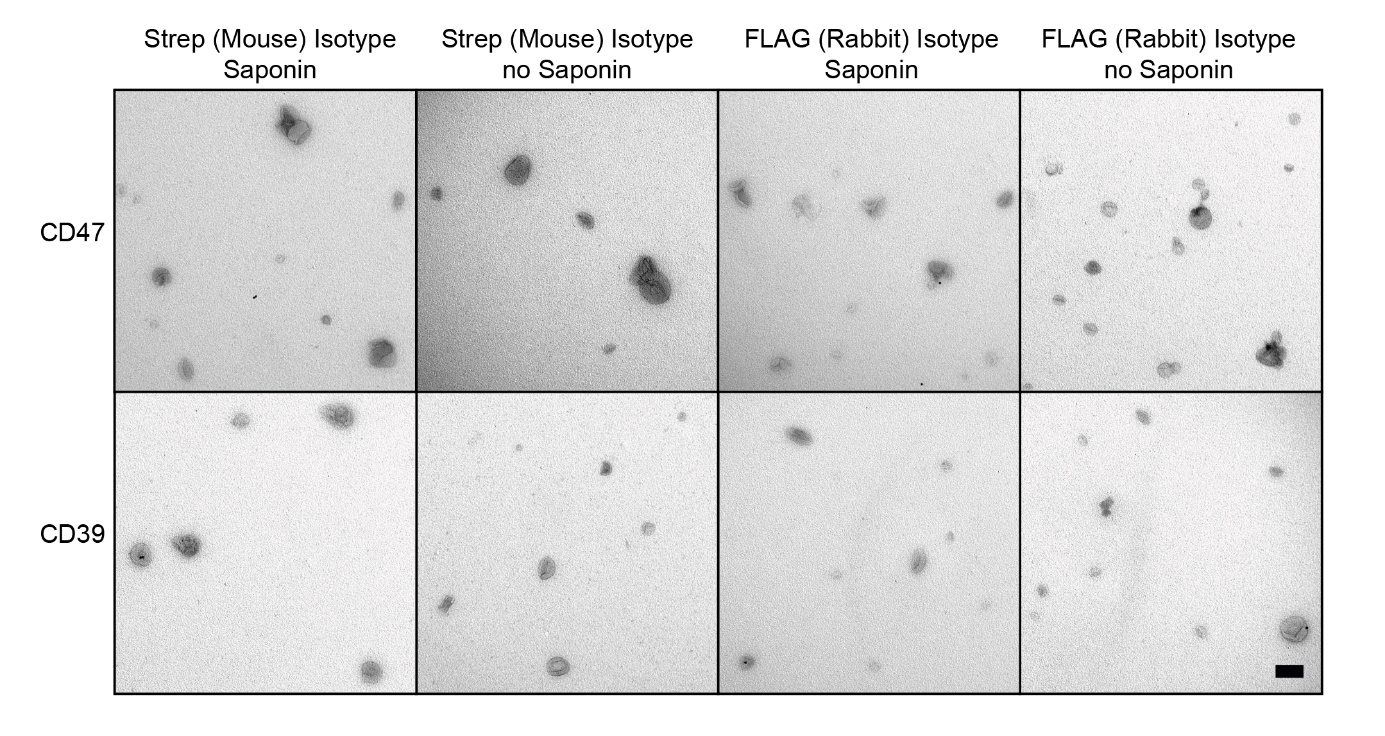


**Figure S1: Isotype-labeled immuno-electron microscopy images of CD47 EV mimetics and CD39 EV mimetics.** Labeling was performed in the absence and presence of saponin. Scale bar: 200 nm.


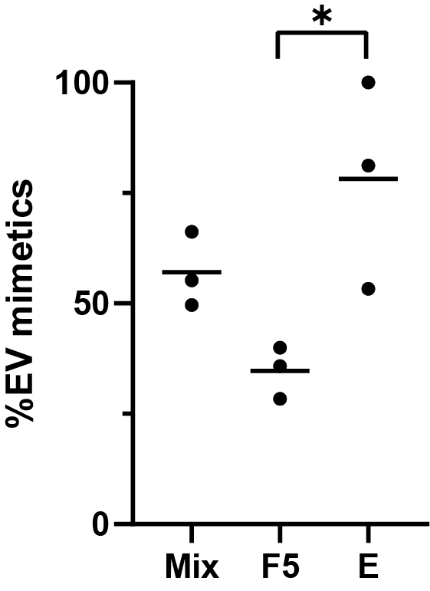


**Figure S2: Strep-tag affinity purification allows quantitative enrichment of EV mimetics.**  Data is represented as mean of n=3 biological replicates. Each dot represents the mean of 3 individually analyzed immuno-electron microscopy images. One-way ANOVA Tukey’s multiple comparison test was used for statistical analysis, * p<0.05.

**
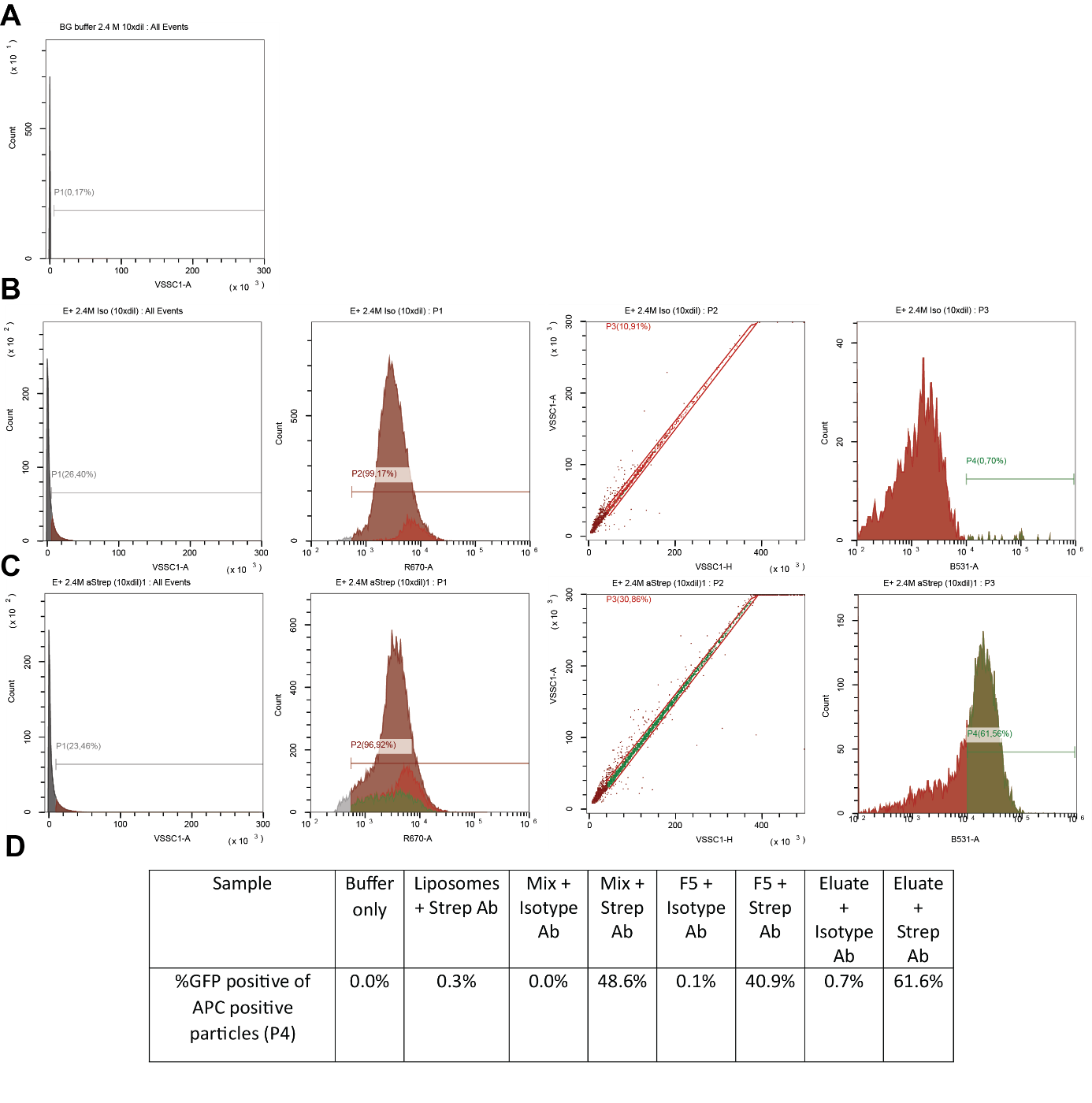
**

**Figure S3: Nano flow cytometric analysis demonstrates successful purification of EV mimetics.** A,B,C: Gating strategy adopted for nano flow cytometry measurements. Particles were first distinguished from buffer background (P1), gated based on their Cy5 fluorescent lipid signal (P2), gated for single particles (P3), and finally the percentage of AF488 positive particles was determined (P4). A: Buffer only, B: Eluate with isotype antibody labeling, C: Eluate with Strep-tag antibody labeling. D: Results of nano flow cytometry measurements.


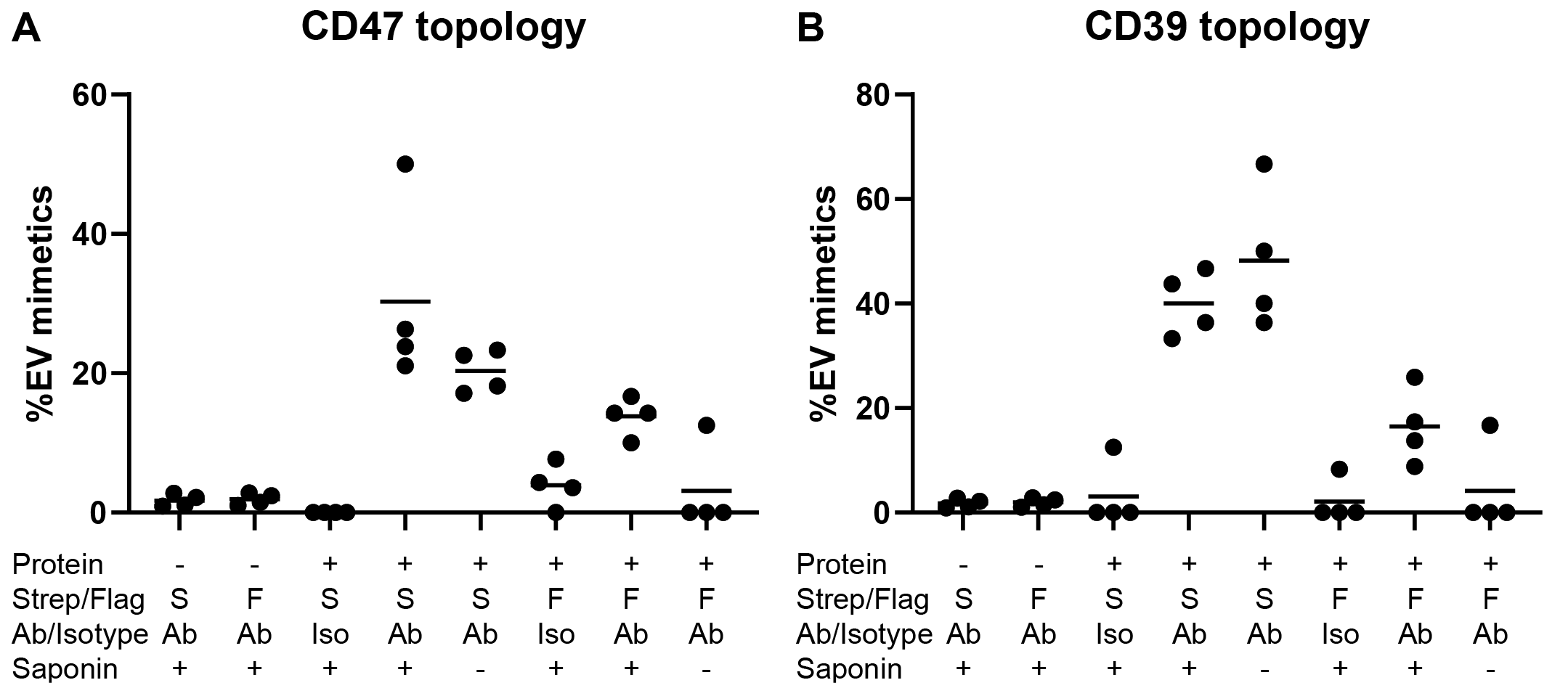


**Figure S4: Quantification of percentage of labeled liposomes using immuno-electron microscopy.** A: CD47 topology, B: CD39 topology. Data is represented as mean of n=4 individually analyzed images.


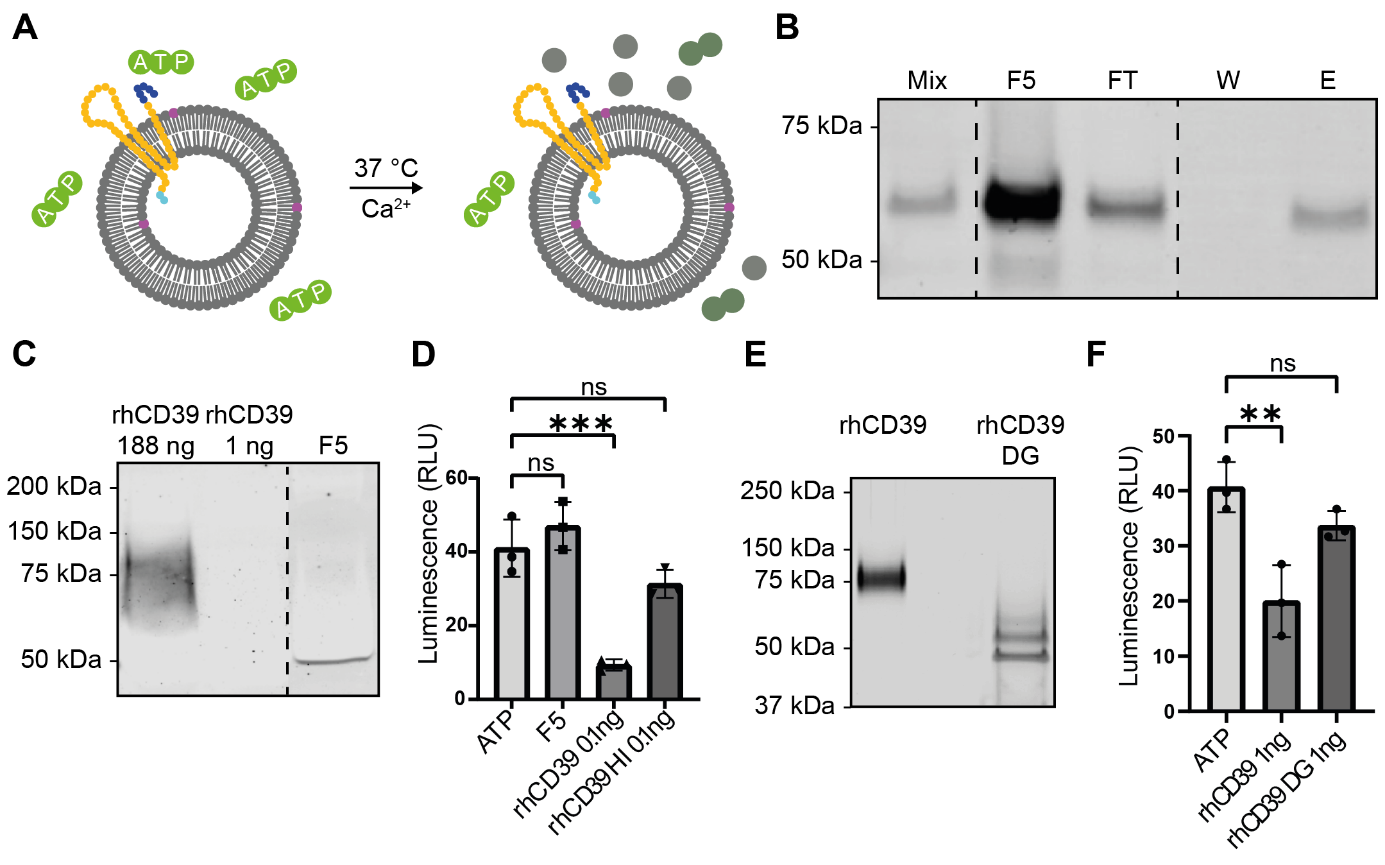


**Figure S5: CD39 EV mimetics show no enzymatic conversion of ATP.** A: Schematic overview of conversion of ATP by CD39 EV mimetics. Enzymatic activity was monitored by measuring ATP breakdown upon incubation of the sample at 37 °C in the presence of Ca^2+^. B: Strep-tag labeled western blot of samples collected at various stages of the purification process of CD39 EV mimetics. Excipients were excluded in this experiment. C: Strep-tag labeled western blot of recombinant human CD39 (rhCD39) and CD39 EV mimetics upon sucrose density gradient ultracentrifugation (DGU) (F5). D: ATP detection assay of CD39 EV mimetics upon DGU (F5) and rhCD39. Additionally, rhCD39 enzymatic activity was assessed upon heat-inactivation (HI). E: Coomassie blue staining of untreated rhCD39 and rhCD39 upon deglycosylation (DG). F: ATP detection assay of rhCD39 and rhCD39 upon DG. One-way ANOVA Tukey’s multiple comparison test was used for statistical analysis, ** p<0.001, *** p<0.0001, individual values ± SD are displayed.


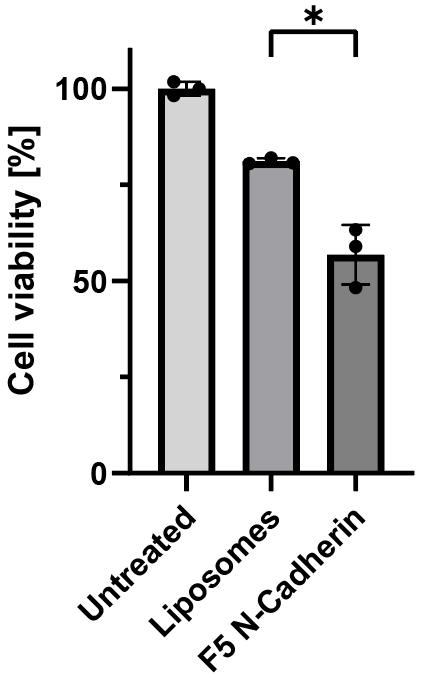


**Figure S6: Quantification of cell viability upon treatment of MDA-MB-231 cells with paclitaxel-loaded liposomes or paclitaxel-loaded N-Cadherin EV mimetics.** Welch’s students t-test was used for statistical analysis, * p<0.05, individual values ± SD are displayed.


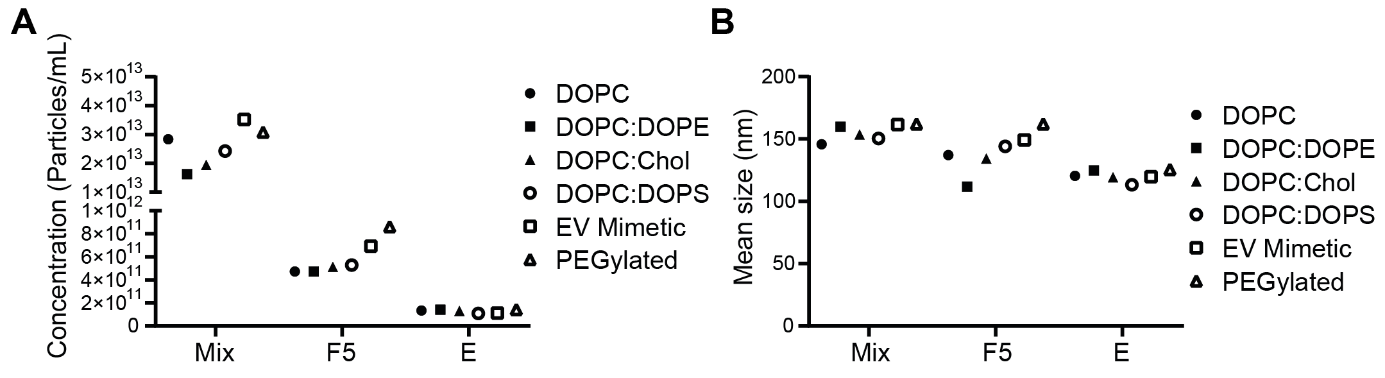


**Figure S7:** **Size & concentration characterization of EV mimetics prepared using various lipid compositions.** A: Nanoparticle Tracking Analysis representing concentration of each liposomal formulation upon incubating in cell-free protein synthesis reaction (Mix), density gradient ultracentrifugation (DGU) (F5), and upon affinity-based purification (E). B: Nanoparticle Tracking Analysis representing mean size of each liposomal formulation upon incubating in cell-free protein synthesis reaction (Mix), DGU (F5), and purification (E).


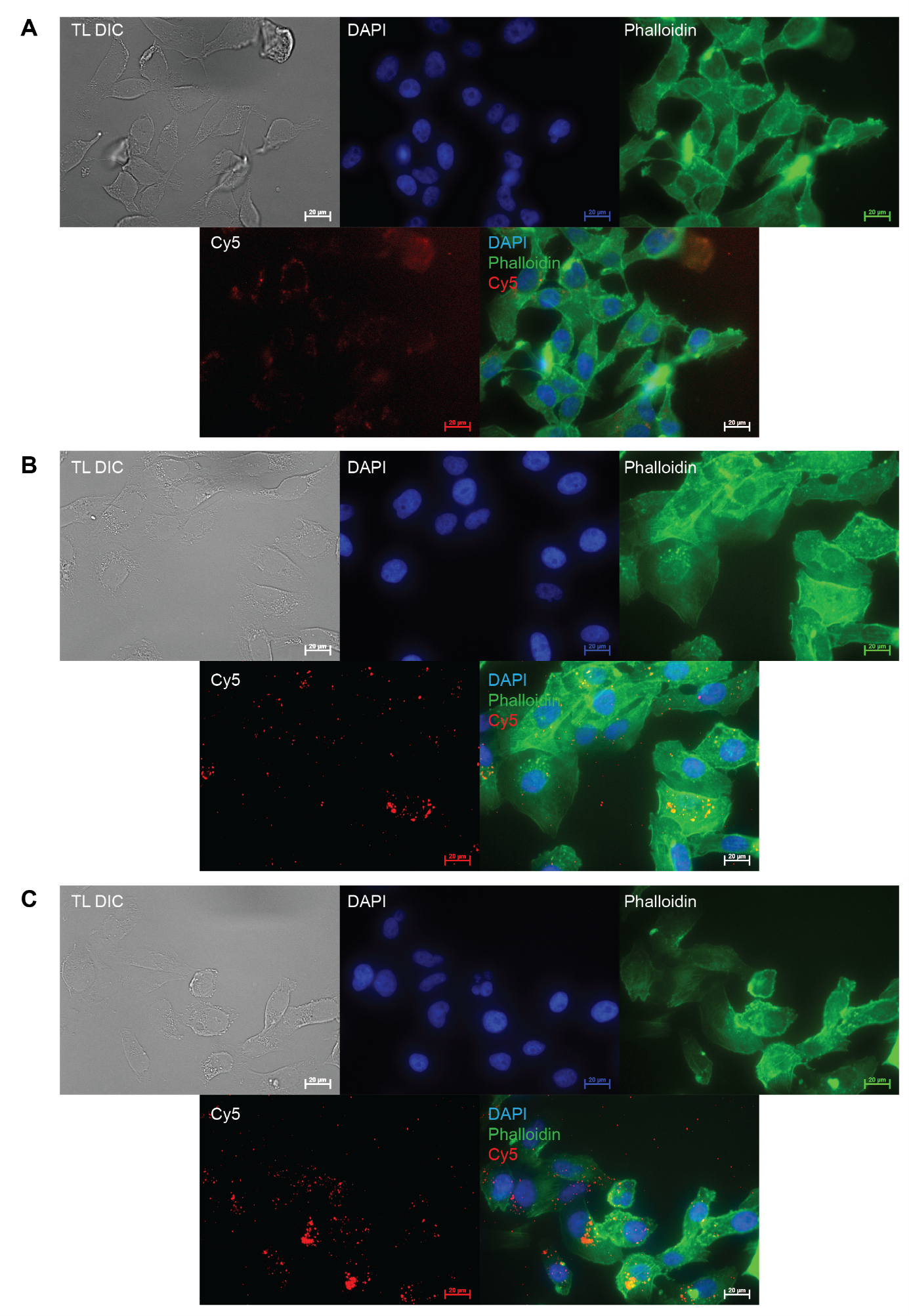


**Figure S8: Fluorescence microscopy images of MDA-MB-231 cells treated with fluorescent liposomes and N-Cadherin EV mimetics.** A: Untreated, B: Liposome treated, C: N-Cadherin EV mimetic treated. Scale bar: 20 µm.

**
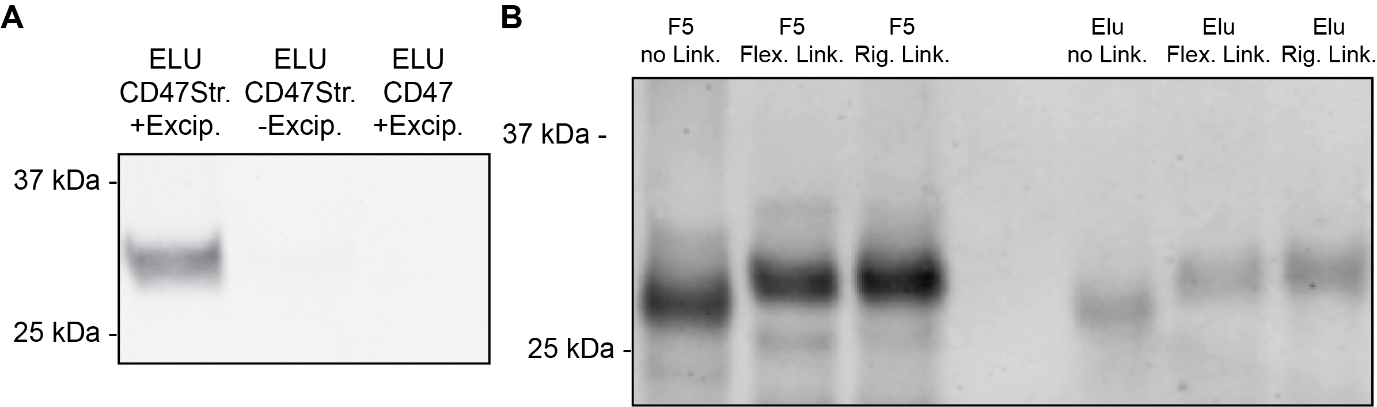
**

**Figure S9: Optimizations for enhanced EV mimetic yield.** A: Strep-tag labeled western blot showing the effect of addition of excipients on EV mimetic yield in the eluate upon purification using Streptactin XT resin. The excipients consist of 0.2% bovine serum albumin, 25 mM Trehalose, and protease inhibitors. B: Strep-tag labeled western blot showing the effect of a flexible (GGGGS)_5_ or rigid A(EAAAK)_5_A amino acid linker between N-terminal Twin-Strep-tag and CD47. Samples are shown before (F5) and after purification (Elu).

**Table S1: Overview of GBlock constructs used in this work, along with restriction sites.**

| **CD47** (NdeI and BamHI) |
| --- |
| **DNA sequence 5’3’** |
| CAT ATG TGG CCA CTG GTT GCG GCC CTT CTG TTG GGG TCA GCG TGC TGT GGT AGC GCA CAA CTT TTG TTC AAT AAA ACT AAG TCA GTG GAA TTT ACT TTT TGC AAC GAT ACA GTA GTA ATT CCC TGT TTT GTA ACT AAC ATG GAA GCC CAA AAC ACA ACA GAA GTC TAT GTC AAA TGG AAA TTC AAG GGC CGT GAT ATT TAC ACG TTC GAC GGA GCC CTG AAC AAG AGC ACC GTG CCG ACC GAC TTC TCC AGT GCT AAA ATC GAG GTT TCC CAA CTT CTT AAG GGG GAT GCA TCT CTT AAG ATG GAT AAG TCA GAC GCT GTA TCT CAC ACG GGT AAT TAT ACC TGC GAG GTA ACG GAG CTT ACG CGC GAG GGA GAA ACA ATC ATC GAG TTG AAG TAC CGC GTT GTA AGT TGG TTT TCT CCG AAC GAG AAC ATC TTG ATT GTC ATC TTT CCC ATT TTT GCT ATC CTG CTG TTT TGG GGT CAG TTT GGT ATT AAG ACA TTG AAA TAC CGC TCC GGG GGT ATG GAC GAA AAG ACA ATT GCT TTG CTG GTA GCC GGA TTG GTC ATC ACC GTT ATC GTG ATC GTG GGA GCA ATT CTT TTT GTG CCC GGC GAG TAC TCG CTT AAG AAC GCC ACT GGA TTA GGT CTG ATC GTA ACT TCC ACT GG CAT TCT GAT TTT ATT GCA CTA TTA TGT ATT CTC GAC AGC AAT CGG GTT GAC CTC GTT TGT AAT CGC CAT TTT GGT CAT TCA AGT AAT TGC TTAT ATT CTG GCG GTA GTA GGG TTG TCC TTG TGT ATT GCC GCC TGC ATC CCC ATG CAT GGC CCA TTA CTT ATC TCG GGG CTT TCA ATC TTA GCT TTA GCG CAG CTT CTG GGA TTA GTG TAT ATG AAG TTT GTT GCT TCT AAT CAA AAA ACA ATT CAG CCA CCT CGC AAG GCC GTT GAG GAA CCC TTG AAT GCA TTC AAG GAA TCG AAG GGC ATG ATG AAC GAT GAA TAA TGA GGA TCC |
| **CD47, containing an N-terminal Twin-Strep-tag sequence** (NdeI and BamHI) |
| **DNA sequence 5’3’** |
| CAT ATG GCC AGC GCT TGG TCT CAT CCT CAG TTC GAG AAG GGT GGG GGA TCA GGG GGA GGG TCC GGC GGG TCT GCA TGG AGC CAT CCA CAG TTT GAA AAA TCC GCT TGG CCA CTG GTT GCG GCC CTT CTG TTG GGG TCA GCG TGC TGT GGT AGC GCA CAA CTT TTG TTC AAT AAA ACT AAG TCA GTG GAA TTT ACT TTT TGC AAC GAT ACA GTA GTA ATT CCC TGT TTT GTA ACT AAC ATG GAA GCC CAA AAC ACA ACA GAA GTC TAT GTC AAA TGG AAA TTC AAG GGC CGT GAT ATT TAC ACG TTC GAC GGA GCC CTG AAC AAG AGC ACC GTG CCG ACC GAC TTC TCC AGT GCT AAA ATC GAG GTT TCC CAA CTT CTT AAG GGG GAT GCA TCT CTT AAG ATG GAT AAG TCA GAC GCT GTA TCT CAC ACG GGT AAT TAT ACC TGC GAG GTA ACG GAG CTT ACG CGC GAG GGA GAA ACA ATC ATC GAG TTG AAG TAC CGC GTT GTA AGT TGG TTT TCT CCG AAC GAG AAC ATC TTG ATT GTC ATC TTT CCC ATT TTT GCT ATC CTG CTG TTT TGG GGT CAG TTT GGT ATT AAG ACA TTG AAA TAC CGC TCC GGG GGT ATG GAC GAA AAG ACA ATT GCT TTG CTG GTA GCC GGA TTG GTC ATC ACC GTT ATC GTG ATC GTG GGA GCA ATT CTT TTT GTG CCC GGC GAG TAC TCG CTT AAG AAC GCC ACT GGA TTA GGT CTG ATC GTA ACT TCC ACT GGC ATT CTG ATT TTA TTG CAC TAT TAT GTA TTC TCG ACA GCA ATC GGG TTG ACC TCG TTT GTA ATC GCC ATT TTG GTC ATT CAA GTA ATT GCT TAT ATT CTG GCG GTA GTA GGG TTG TCC TTG TGT ATT GCC GCC TGC ATC CCC ATG CAT GGC CCA TTA CTT ATC TCG GGG CTT TCA ATC TTA GCT TTA GCG CAG CTT CTG GGA TTA GTG TAT ATG AAG TTT GTT GCT TCT AAT CAA AAA ACA ATT CAG CCA CCT CGC AAG GCC GTT GAG GAA CCC TTG AAT GCA TTC AAG GAA TCG AAG GGC ATG ATG AAC GAT GAA TAA TGA GGA TCC |
| **CD47, containing an N-terminal Twin-Strep-tag sequence and a Flexible (GGGGS)_5_ linker Between N-terminal Twin-Strep-tag and CD47** (NdeI and BamHI) |
| **DNA sequence 5’3’** |
| CAT ATG TCA GCT TGG AGT CAC CCA CAG TTC GAG AAA GGG GGC GGG AGT GGG GGA GGT TCT GGC GGG AGC GCC TGG TCT CAT CCC CAG TTT GAG AAG GGA GGA GGA GGT AGT GGT GGC GGC GGA TCG GGA GGT GGC GGT TCA GGT GGT GGT GGA TCT GGT GGA GGT GGA TCA GGT ACC TGG CCA CTG GTT GCG GCC CTT CTG TTG GGG TCA GCG TGC TGT GGT AGC GCA CAA CTT TTG TTC AAT AAA ACT AAG TCA GTG GAA TTT ACT TTT TGC AAC GAT ACA GTA GTA ATT CCC TGT TTT GTA ACT AAC ATG GAA GCC CAA AAC ACA ACA GAA GTC TAT GTC AAA TGG AAA TTC AAG GGC CGT GAT ATT TAC ACG TTC GAC GGA GCC CTG AAC AAG AGC ACC GTG CCG ACC GAC TTC TCC AGT GCT AAA ATC GAG GTT TCC CAA CTT CTT AAG GGG GAT GCA TCT CTT AAG ATG GAT AAG TCA GAC GCT GTA TCT CAC ACG GGT AAT TAT ACC TGC GAG GTA ACG GAG CTT ACG CGC GAG GGA GAA ACA ATC ATC GAG TTG AAG TAC CGC GTT GTA AGT TGG TTT TCT CCG AAC GAG AAC ATC TTG ATT GTC ATC TTT CCC ATT TTT GCT ATC CTG CTG TTT TGG GGT CAG TTT GGT ATT AAG ACA TTG AAA TAC CGC TCC GGG GGT ATG GAC GAA AAG ACA ATT GCT TTG CTG GTA GCC GGA TTG GTC ATC ACC GTT ATC GTG ATC GTG GGA GCA ATT CTT TTT GTG CCC GGC GAG TAC TCG CTT AAG AAC GCC ACT GGA TTA GGT CTG ATC GTA ACT TCC ACT GGC ATT CTG ATT TTA TTG CAC TAT TAT GTA TTC TCG ACA GCA ATC GGG TTG ACC TCG TTT GTA ATC GCC ATT TTG GTC ATT CAA GTA ATT GCT TAT ATT CTG GCG GTA GTA GGG TTG TCC TTG TGT ATT GCC GCC TGC ATC CCC ATG CAT GGC CCA TTA CTT ATC TCG GGG CTT TCA ATC TTA GCT TTA GCG CAG CTT CTG GGA TTA GTG TAT ATG AAG TTT GTT GCT TCT AAT CAA AAA ACA ATT CAG CCA CCT CGC AAG GCC GTT GAG GAA CCC TTG AAT GCA TTC AAG GAA TCG AAG GGC ATG ATG AAC GAT GAA TAA GGA TCC |
| **CD47, containing an N-terminal Twin-Strep-tag sequence and a Rigid A(EAAAK)_5_A linker Between N-terminal Twin-Strep-tag and CD47** (NdeI and BamHI) |
| **DNA sequence 5’3’** |
| CAT ATG AGC GCA TGG AGT CAT CCG CAG TTC GAA AAA GGA GGA GGA AGT GGA GGT GGA TCT GGC GGG AGT GCA TGG TCA CAT CCA CAG TTC GAG AAG GCA GAG GCG GCT GCG AAA GAA GCA GCC GCC AAG GAA GCA GCT GCA AAA GAG GCT GCT GCT AAG GAG GCG GCT GCG AAG GCG GGT ACC CAA CTT TTG TTC AAT AAA ACT AAG TCA GTG GAA TTT ACT TTT TGC AAC GAT ACA GTA GTA ATT CCC TGT TTT GTA ACT AAC ATG GAA GCC CAA AAC ACA ACA GAA GTC TAT GTC AAA TGG AAA TTC AAG GGC CGT GAT ATT TAC ACG TTC GAC GGA GCC CTG AAC AAG AGC ACC GTG CCG ACC GAC TTC TCC AGT GCT AAA ATC GAG GTT TCC CAA CTT CTT AAG GGG GAT GCA TCT CTT AAG ATG GAT AAG TCA GAC GCT GTA TCT CAC ACG GGT AAT TAT ACC TGC GAG GTA ACG GAG CTT ACG CGC GAG GGA GAA ACA ATC ATC GAG TTG AAG TAC CGC GTT GTA AGT TGG TTT TCT CCG AAC GAG AAC ATC TTG ATT GTC ATC TTT CCC ATT TTT GCT ATC CTG CTG TTT TGG GGT CAG TTT GGT ATT AAG ACA TTG AAA TAC CGC TCC GGG GGT ATG GAC GAA AAG ACA ATT GCT TTG CTG GTA GCC GGA TTG GTC ATC ACC GTT ATC GTG ATC GTG GGA GCA ATT CTT TTT GTG CCC GGC GAG TAC TCG CTT AAG AAC GCC ACT GGA TTA GGT CTG ATC GTA ACT TCC ACT GGC ATT CTG ATT TTA TTG CAC TAT TAT GTA TTC TCG ACA GCA ATC GGG TTG ACC TCG TTT GTA ATC GCC ATT TTG GTC ATT CAA GTA ATT GCT TAT ATT CTG GCG GTA GTA GGG TTG TCC TTG TGT ATT GCC GCC TGC ATC CCC ATG CAT GGC CCA TTA CTT ATC TCG GGG CTT TCA ATC TTA GCT TTA GCG CAG CTT CTG GGA TTA GTG TAT ATG AAG TTT GTT GCT TCT AAT CAA AAA ACA ATT CAG CCA CCT CGC AAG GCC GTT GAG GAA CCC TTG AAT GCA TTC AAG GAA TCG AAG GGC ATG ATG AAC GAT GAA TAA GGA TCC |
| **CD47, containing an N-terminal Twin-Strep-tag and a C-terminal FLAG-tag sequence** (NdeI and BamHI) |
| **DNA sequence 5’3’** |
| CAT ATG GCC TGG TCC CAC CCT CAG TTT GAA AAG GGC GGA GGT AGC GGT GGC GGT TCA GGT GGG TCA GCG TGG TCT CAT CCC CAG TTC GAA AAG GGT ACC TGG CCA CTG GTT GCG GCC CTT CTG TTG GGG TCA GCG TGC TGT GGT AGC GCA CAA CTT TTG TTC AAT AAA ACT AAG TCA GTG GAA TTT ACT TTT TGC AAC GAT ACA GTA GTA ATT CCC TGT TTT GTA ACT AAC ATG GAA GCC CAA AAC ACA ACA GAA GTC TAT GTC AAA TGG AAA TTC AAG GGC CGT GAT ATT TAC ACG TTC GAC GGA GCC CTG AAC AAG AGC ACC GTG CCG ACC GAC TTC TCC AGT GCT AAA ATC GAG GTT TCC CAA CTT CTT AAG GGG GAT GCA TCT CTT AAG ATG GAT AAG TCA GAC GCT GTA TCT CAC ACG GGT AAT TAT ACC TGC GAG GTA ACG GAG CTT ACG CGC GAG GGA GAA ACA ATC ATC GAG TTG AAG TAC CGC GTT GTA AGT TGG TTT TCT CCG AAC GAG AAC ATC TTG ATT GTC ATC TTT CCC ATT TTT GCT ATC CTG CTG TTT TGG GGT CAG TTT GGT ATT AAG ACA TTG AAA TAC CGC TCC GGG GGT ATG GAC GAA AAG ACA ATT GCT TTG CTG GTA GCC GGA TTG GTC ATC ACC GTT ATC GTG ATC GTG GGA GCA ATT CTT TTT GTG CCC GGC GAG TAC TCG CTT AAG AAC GCC ACT GGA TTA GGT CTG ATC GTA ACT TCC ACT GGC ATT CTG ATT TTA TTG CAC TAT TAT GTA TTC TCG ACA GCA ATC GGG TTG ACC TCG TTT GTA ATC GCC ATT TTG GTC ATT CAA GTA ATT GCT TAT ATT CTG GCG GTA GTA GGG TTG TCC TTG TGT ATT GCC GCC TGC ATC CCC ATG CAT GGC CCA TTA CTT ATC TCG GGG CTT TCA ATC TTA GCT TTA GCG CAG CTT CTG GGA TTA GTG TAT ATG AAG TTT GTT GCT TCT AAT CAA AAA ACA ATT CAG CCA CCT CGC AAG GCC GTT GAG GAA CCC TTG AAT GCA TTC AAG GAA TCG AAG GGC ATG ATG AAC GAT GAA GAT TAC AAG GAT GAT GAC GAT AAG TAA GGA TCC |
| **CD39, containing an N-terminal FLAG-tag and a C-terminal SnorkelTag & Twin-Strep-tag sequence** (NdeI and BamHI) |
| **DNA sequence 5’3’** |
| CAT ATG GAT TAC AAA GAT GAT GAC GAT AAA GGT GGC GGA GGG TCA GAA GAC ACG AAG GAA TCA AAC GTC AAA ACC TTT TGT TCG AAG AAC ATC CTG GCG ATT TTG GGA TTT TCA AGT ATC ATT GCC GTA ATT GCC CTT TTA GCT GTC GGG CTT ACT CAG AAT AAA GCT CTG CCA GAG AAC GTC AAG TAC GGA ATT GTT CTG GAT GCC GGG TCG TCG CAC ACG TCC CTG TAT ATT TAT AAA TGG CCC GCA GAG AAG GAA AAC GAT ACA GGA GTT GTC CAC CAG GTG GAA GAA TGC CGT GTA AAG GGG CCA GGG ATC TCA AAG TTC GTA CAG AAA GTA AAC GAA ATC GGA ATT TAT CTG ACC GAT TGC ATG GAG CGC GCA CGT GAA GTT ATC CCA CGC TCA CAG CAC CAA GAA ACT CCT GTG TAT CTG GGT GCA ACC GCA GGC ATG CGT TTA CTT CGC ATG GAG TCT GAG GAA CTT GCC GAC CGT GTT TTG GAT GTC GTT GAA CGC AGT CTT TCA AAT TAC CCC TTC GAT TTC CAG GGT GCA CGT ATC ATC ACC GGG CAG GAG GAG GGT GCT TAT GGA TGG ATT ACG ATT AAT TAT CTT CTT GGT AAG TTT TCC CAA AAA ACT CGT TGG TTC TCC ATT GTT CCC TAT GAA ACG AAT AAC CAG GAA ACA TTC GGA GCC CTT GAT TTA GGT GGT GCC TCA ACC CAA GTT ACA TTC GTA CCG CAA AAT CAG ACC ATC GAG TCG CCG GAT AAC GCG TTG CAG TTT CGT TTA TAT GGG AAG GAT TAT AAT GTG TAT ACA CAT AGT TTT CTT TGC TAT GGT AAG GAT CAG GCT CTG TGG CAA AAG CTG GCA AAA GAT ATT CAA GTT GCG AGT AAC GAG ATC TTA CGC GAT CCT TGT TTT CAT CCA GGT TAT AAA AAA GTA GTA AAC GTC AGC GAC TTA TAC AAA ACA CCT TGC ACT AAG CGT TTT GAG ATG ACT TTG CCA TTT CAG CAG TTT GAG ATT CAA GGC ATT GGC AAT TAT CAG CAG TGC CAT CAA AGC ATC CTT GAG CTT TTT AAC ACT TCT TAC TGT CCG TAC AGC CAA TGT GCC TTC AAT GGC ATT TTT TTG CCA CCA TTG CAA GGA GAC TTC GGT GCC TTC TCG GCG TTC TAC TTC GTT ATG AAG TTC CTG AAT TTA ACC TCC GAA AAA GTA AGT CAA GAA AAA GTC ACA GAA ATG ATG AAA AAG TTT TGT GCG CAG CCG TGG GAA GAG ATC AAG ACA TCG TAT GCG GGA GTT AAG GAA AAG TAC TTG TCA GAG TAC TGT TTC TCA GGA ACG TAC ATC TTA TCC CTG TTA CTT CAA GGT TAT CAC TTT ACC GCC GAT TCC TGG GAA CAT ATC CAC TTT ATC GGA AAG ATC CAA GGG TCG GAC GCG GGC TGG ACT CTG GGG TAT ATG CTG AAT CTT ACG AAT ATG ATT CCT GCA GAA CAA CCG CTT TCT ACG CCA TTG TCA CAC AGT ACG TAT GTT TTC TTG ATG GTA TTA TTC TCA CTG GTT TTA TTT ACC GTT GCG ATT ATC GGG TTG TTA ATT TTC CAT AAA CCA TCA TAC TTC TGG AAG GAT ATG GTA GGA GCC TCC TCC GGC AGC TCT GGA AGC GGC TCA CAA AAG AAA CCC CGT TAT GAG ATC CGC TGG AAA GTC GTC GTT ATC AGT GCA ATC TTA GCC CTG GTC GTT TTG ACC GTG ATT TCA CTT ATC ATC CTT ATC ATG TTA TGG GGC GGG GGT GGA TCT TCG GCT TGG TCA CAC CCG CAA TTT GAG AAA GGG GGA GGA TCG GGC GGC GGG TCC GGT GGC TCA GCG TGG TCG CAT CCG CAG TTC GAG AAA TGA GGA TCC |
| **N-Cadherin, containing a C-terminal SnorkelTag & Twin-Strep-tag sequence** (NdeI and BamHI) |
| **DNA sequence 5’3’** |
| CAT ATG GAC TGG GTC ATC CCC CCC ATC AAC CTT CCC GAG AAC TCG CGT GGT CCC TTC CCA CAA GAA CTG GTA CGC ATC CGT TCT GAC CGT GAT AAA AAC CTT TCG TTA CGT TAC TCC GTG ACT GGT CCG GGT GCC GAC CAG CCG CCC ACG GGT ATT TTC ATC ATT AAT CCA ATC AGC GGG CAG CTG TCG GTG ACA AAG CCT TTG GAC CGT GAG CAA ATT GCA CGC TTT CAT CTT CGC GCA CAC GCA GTG GAT ATT AAT GGT AAT CAA GTT GAG AAC CCA ATT GAT ATC GTA ATT AAC GTC ATT GAT ATG AAT GAT AAT CGC CCG GAG TTC TTG CAC CAG GTC TGG AAC GGC ACC GTA CCG GAA GGG TCC AAG CCC GGG ACA TAC GTT ATG ACG GTA ACT GCT ATT GAT GCC GAT GAC CCT AAC GCG TTG AAT GGA ATG CTT CGC TAT CGT ATT GTA TCG CAG GCC CCT TCA ACT CCC AGT CCC AAC ATG TTT ACT ATC AAT AAT GAA ACA GGC GAC ATT ATC ACT GTT GCG GCG GGG TTG GAT CGT GAG AAG GTA CAA CAA TAC ACT CTT ATC ATC CAG GCG ACG GAT ATG GAA GGT AAT CCT ACG TAC GGC TTG AGT AAT ACA GCC ACA GCG GTT ATC ACC GTT ACC GAC GTG AAC GAC AAC CCT CCG GAA TTC ACT GCA ATG ACC TTC TAT GGG GAG GTC CCG GAG AAT CGT GTG GAT ATC ATC GTG GCA AAT TTG ACT GTG ACT GAT AAG GAT CAA CCT CAC ACC CCG GCA TGG AAC GCT GTT TAC CGT ATC TCA GGA GGG GAC CCA ACC GGC CGC TTC GCG ATT CAG ACG GAC CCT AAT TCT AAC GAC GGC CTG GTA ACG GTG GTG AAG CCG ATT GAT TTC GAG ACC AAT CGC ATG TTC GTA CTT ACC GTG GCA GCG GAA AAC CAG GTC CCC TTA GCT AAA GGC ATC CAA CAC CCT CCA CAG AGT ACA GCT ACA GTC AGC GTT ACG GTG ATC GAT GTG AAC GAA AAT CCG TAC TTC GCG CCC AAC CCC AAA ATC ATC CGT CAA GAA GAG GGT CTT CAC GCA GGG ACC ATG TTA ACC ACA TTC ACT GCG CAG GAC CCA GAT CGC TAT ATG CAA CAG AAT ATT CGT TAT ACA AAG CTG AGC GAT CCG GCT AAT TGG TTG AAG ATT GAC CCC GTC AAT GGA CAA ATT ACC ACC ATT GCT GTG CTT GAT CGT GAG AGC CCT AAT GTT AAG AAT AAC ATC TAT AAC GCA ACA TTC TTA GCT AGT GAT AAT GGA ATT CCC CCC ATG TCG GGC ACA GGG ACT TTG CAG ATT TAC TTA CTG GAT ATT AAT GAT AAT GCA CCA CAG GTC TTG CCG CAG GAA GCA GAA ACC TGC GAA ACT CCA GAC CCT AAC TCT ATT AAC ATC ACT GCT CTG GAC TAC GAT ATC GAT CCC AAT GCA GGC CCC TTT GCT TTT GAC CTG CCC TTG TCA CCG GTT ACC ATT AAA CGT AAC TGG ACA ATT ACG CGT CTT AAC GGC GAT TTC GCC CAG TTA AAC TTG AAA ATT AAG TTC CTG GAA GCG GGT ATC TAT GAA GTT CCA ATT ATT ATC ACT GAT TCT GGC AAT CCC CCT AAG AGC AAC ATC TCC ATT CTT CGT GTG AAG GTT TGC CAA TGC GAC AGT AAC GGG GAC TGC ACA GAT GTT GAT CGT ATT GTG GGA GCA GGG TTA GGG ACT GGG GCG ATT ATC GCC ATT CTG TTG TGT ATT ATC ATT CTG TTG ATT CTT GTA TTG ATG TTT GTT GTT TGG ATG AAA CGT CGT GAT AAA GAA CGC CAA GCC AAG CAA CTG CTG ATC GAC CCA GAG GAC GAT GTC CGT GAT AAT ATC TTG AAG TAC GAT GAA GAG GGT GGA GGA GAG GAG GAC CAA GAC TAC GAC CTG TCC CAA TTG CAA CAG CCA GAC ACA GTT GAA CCA GAT GCT ATC AAA CCG GTG GGT ATT CGC CGC ATG GAC GAA CGC CCA ATC CAT GCG GAA CCA CAG TAC CCA GTA CGC AGC GCA GCA CCG CAC CCG GGG GAC ATC GGA GAC TTC ATC AAC GAG GGA TTA AAA GCT GCA GAT AAC GAC CCA ACC GCT CCA CCG TAC GAT TCG CTG CTG GTA TTC GAT TAC GAA GGT AGC GGT AGC ACT GCA GGG TCT TTA TCT AGT CTG AAT TCG TCC TCG TCT GGG GGG GAG CAG GAC TAT GAC TAC TTA AAC GAC TGG GGG CCA CGC TTC AAA AAA TTG GCC GAT ATG TAT GGA GGT GGA GAC GAC GGT ACC GGT GCA AGC TCG GGG TCG AGC GGT TCC GGC TCG CAA AAG AAA CCG CGC TAT GAA ATT CGC TGG AAG GTC GTG GTG ATC TCG GCG ATC TTA GCA TTA GTC GTG TTA ACA GTG ATT TCC TTG ATT ATC TTG ATT ATG CTG TGG GGC GGA GGG GGT TCG AGC GCA TGG TCC CAT CCC CAG TTC GAG AAA GGA GGA GGT TCC GGC GGT GGG TCA GGC GGC TCG GCT TGG TCT CAC CCT CAG TTC GAA AAA TGA GGA TCC |
| **N-Cadherin mutant (D134A)** (NdeI and BamHI) |
| **DNA sequence 5’3’** |
| CAT ATG GAC TGG GTC ATC CCC CCC ATC AAC CTT CCC GAG AAC TCG CGT GGT CCC TTC CCA CAA GAA CTG GTA CGC ATC CGT TCT GAC CGT GAT AAA AAC CTT TCG TTA CGT TAC TCC GTG ACT GGT CCG GGT GCC GAC CAG CCG CCC ACG GGT ATT TTC ATC ATT AAT CCA ATC AGC GGG CAG CTG TCG GTG ACA AAG CCT TTG GAC CGT GAG CAA ATT GCA CGC TTT CAT CTT CGC GCA CAC GCA GTG GAT ATT AAT GGT AAT CAA GTT GAG AAC CCA ATT GAT ATC GTA ATT AAC GTC ATT GAT ATG AAT GAT AAT CGC CCG GAG TTC TTG CAC CAG GTC TGG AAC GGC ACC GTA CCG GAA GGG TCC AAG CCC GGG ACA TAC GTT ATG ACG GTA ACT GCT ATT GCA GCC GAT GAC CCT AAC GCG TTG AAT GGA ATG CTT CGC TAT CGT ATT GTA TCG CAG GCC CCT TCA ACT CCC AGT CCC AAC ATG TTT ACT ATC AAT AAT GAA ACA GGC GAC ATT ATC ACT GTT GCG GCG GGG TTG GAT CGT GAG AAG GTA CAA CAA TAC ACT CTT ATC ATC CAG GCG ACG GAT ATG GAA GGT AAT CCT ACG TAC GGC TTG AGT AAT ACA GCC ACA GCG GTT ATC ACC GTT ACC GAC GTG AAC GAC AAC CCT CCG GAA TTC ACT GCA ATG ACC TTC TAT GGG GAG GTC CCG GAG AAT CGT GTG GAT ATC ATC GTG GCA AAT TTG ACT GTG ACT GAT AAG GAT CAA CCT CAC ACC CCG GCA TGG AAC GCT GTT TAC CGT ATC TCA GGA GGG GAC CCA ACC GGC CGC TTC GCG ATT CAG ACG GAC CCT AAT TCT AAC GAC GGC CTG GTA ACG GTG GTG AAG CCG ATT GAT TTC GAG ACC AAT CGC ATG TTC GTA CTT ACC GTG GCA GCG GAA AAC CAG GTC CCC TTA GCT AAA GGC ATC CAA CAC CCT CCA CAG AGT ACA GCT ACA GTC AGC GTT ACG GTG ATC GAT GTG AAC GAA AAT CCG TAC TTC GCG CCC AAC CCC AAA ATC ATC CGT CAA GAA GAG GGT CTT CAC GCA GGG ACC ATG TTA ACC ACA TTC ACT GCG CAG GAC CCA GAT CGC TAT ATG CAA CAG AAT ATT CGT TAT ACA AAG CTG AGC GAT CCG GCT AAT TGG TTG AAG ATT GAC CCC GTC AAT GGA CAA ATT ACC ACC ATT GCT GTG CTT GAT CGT GAG AGC CCT AAT GTT AAG AAT AAC ATC TAT AAC GCA ACA TTC TTA GCT AGT GAT AAT GGA ATT CCC CCC ATG TCG GGC ACA GGG ACT TTG CAG ATT TAC TTA CTG GAT ATT AAT GAT AAT GCA CCA CAG GTC TTG CCG CAG GAA GCA GAA ACC TGC GAA ACT CCA GAC CCT AAC TCT ATT AAC ATC ACT GCT CTG GAC TAC GAT ATC GAT CCC AAT GCA GGC CCC TTT GCT TTT GAC CTG CCC TTG TCA CCG GTT ACC ATT AAA CGT AAC TGG ACA ATT ACG CGT CTT AAC GGC GAT TTC GCC CAG TTA AAC TTG AAA ATT AAG TTC CTG GAA GCG GGT ATC TAT GAA GTT CCA ATT ATT ATC ACT GAT TCT GGC AAT CCC CCT AAG AGC AAC ATC TCC ATT CTT CGT GTG AAG GTT TGC CAA TGC GAC AGT AAC GGG GAC TGC ACA GAT GTT GAT CGT ATT GTG GGA GCA GGG TTA GGG ACT GGG GCG ATT ATC GCC ATT CTG TTG TGT ATT ATC ATT CTG TTG ATT CTT GTA TTG ATG TTT GTT GTT TGG ATG AAA CGT CGT GAT AAA GAA CGC CAA GCC AAG CAA CTG CTG ATC GAC CCA GAG GAC GAT GTC CGT GAT AAT ATC TTG AAG TAC GAT GAA GAG GGT GGA GGA GAG GAG GAC CAA GAC TAC GAC CTG TCC CAA TTG CAA CAG CCA GAC ACA GTT GAA CCA GAT GCT ATC AAA CCG GTG GGT ATT CGC CGC ATG GAC GAA CGC CCA ATC CAT GCG GAA CCA CAG TAC CCA GTA CGC AGC GCA GCA CCG CAC CCG GGG GAC ATC GGA GAC TTC ATC AAC GAG GGA TTA AAA GCT GCA GAT AAC GAC CCA ACC GCT CCA CCG TAC GAT TCG CTG CTG GTA TTC GAT TAC GAA GGT AGC GGT AGC ACT GCA GGG TCT TTA TCT AGT CTG AAT TCG TCC TCG TCT GGG GGG GAG CAG GAC TAT GAC TAC TTA AAC GAC TGG GGG CCA CGC TTC AAA AAA TTG GCC GAT ATG TAT GGA GGT GGA GAC GAC GGT ACC GGT GCA AGC TCG GGG TCG AGC GGT TCC GGC TCG CAA AAG AAA CCG CGC TAT GAA ATT CGC TGG AAG GTC GTG GTG ATC TCG GCG ATC TTA GCA TTA GTC GTG TTA ACA GTG ATT TCC TTG ATT ATC TTG ATT ATG CTG TGG GGC GGA GGG GGT TCG AGC GCA TGG TCC CAT CCC CAG TTC GAG AAA GGA GGA GGT TCC GGC GGT GGG TCA GGC GGC TCG GCT TGG TCT CAC CCT CAG TTC GAA AAA TGA GGA TCC |
